# Supplementary material for: Do rapid BMI growth in childhood and early-onset obesity offer cardiometabolic protection to obese adults in mid-life? Analysis of a longitudinal cohort study of Danish men
Source: BMJ Open. 2014 Apr 15;4(4):e004827. doi: 10.1136/bmjopen-2014-004827 (PMC3996819; doi:10.1136/bmjopen-2014-004827)
Supplement: Author's manuscript [file bmjopen-2014-004827.draft_revisions.pdf]

Do rapid BMI growth in childhood and early-onset obesity offer cardiometabolic protection to obese adults in mid-life? Analysis of a prospective cohort study of Danish men

Journal:	*BMJ Open*
Manuscript ID:	bmjopen-2014-004827
Article Type:	Research
Date Submitted by the Author:	09-Jan-2014
Complete List of Authors:	Howe, Laura; University of Bristol, Social Medicine<br>Zimmermann, Esther; Institute of Preventive Medicine,<br>Weiss, Ram; The Hebrew University of Jerusalem, Braun School of Public Health<br>S�rensen, Thorkild; Institute of Preventive Medicine
**Primary Subject Heading**:	Epidemiology
Secondary Subject Heading:	Nutrition and metabolism, Cardiovascular medicine
Keywords:	Hypertension < CARDIOLOGY, Lipid disorders < DIABETES & ENDOCRINOLOGY, EPIDEMIOLOGY, NUTRITION & DIETETICS, PRIMARY CARE, PUBLIC HEALTH

SCHOLARONE™
Manuscripts

For peer review only

'Do rapid BMI growth in childhood and early-onset obesity offer cardiometabolic protection to obese adults in mid-life? Analysis of a prospective cohort study of Danish men'

Laura D Howe*[1], Esther Zimmermann[2], Ram Weiss[3], Thorkild IA Sørensen[2,4]

1 – MRC Integrative Epidemiology Unit at the University of Bristol, School of Social and Community Medicine, University of Bristol, Bristol, UK
2 – Institute of Preventive Medicine, Frederiksberg and Bispebjerg Hospitals, The Capital Region, Copenhagen, Denmark
3 – Braun School of Public Health, The Hebrew University of Jerusalem, Israel
4 - Novo Nordisk Center for Basic Metabolic Research, Faculty of Health and Medical Sciences, University of Copenhagen, Denmark

Key words: cardiovascular diseases, growth, metabolic syndrome, obesity, obese but metabolically healthy, metabolically abnormal but normal weight

*Corresponding author: Oakfield House, Oakfield Grove, Bristol, BS8 2BN, UK. Phone: +44 (0)117 3310134. Email: laura.howe@bristol.ac.uk

Article word length: 3,140

For peer review only

For peer review only - http://bmjopen.bmj.com/site/about/guidelines.xhtml

Abstract

Background: Some obese individuals have no cardiometabolic abnormalities; they are 'metabolically healthy, but obese' (MHO). Similarly, some non-obese individuals have cardiometabolic abnormalities, i.e. 'metabolically at risk, normal weight' (MANW). Previous studies have suggested early-onset obesity may be associated with MHO.

Design: general population prospective cohort study

Setting: Denmark

Objective: To assess whether BMI in childhood and early-onset obesity are associated with MHO.

Participants: From 362,200 young men (mean age 20) examined for Danish National Service between 1943-1977, all obese men (BMI≥31 kg/m^2, N=1,930) were identified along with a random 1% sample of the others (N=3,601). Our analysis includes 2,392 of these men attending a research clinic in mid-life (mean age 42). For 613 of these men, data on childhood BMI is available.

Deisgn: We summarised childhood BMI growth (7-13 years) using a multilevel model. Early-onset obesity was defined as obesity at examination for National Service.

Outcome measurement: We defined metabolic health at the mid-life clinic as non-fasting serum cholesterol <6.6mmol/l, non-fasting glucose <8.39mmol/l and pulse pressure <48mmHg. Participants were categorised into four groups according to their obesity (BMI≥30 kg/m^2) and metabolic health in mid-life.

Results:

297 of 1,097 (27.1%) of obese men were metabolically healthy; 826 of 1,295 (63.8%) non-obese mean had at least one metabolic abnormality. There was no evidence that rapid BMI growth in childhood or early-onset obesity was associated with either MHO or the MANW phenotype, e.g. amongst obese men in mid-life, the odds ratio for MHO comparing early-onset obesity with non-early-onset obesity was 0.97 (95% confidence interval 0.85 to 1.10).

Conclusion: We found no robust evidence that early onset obesity or rapid BMI growth in childhood is protective for cardiometabolic health.

Key words: metabolically healthy but obese, metabolic risk, cardiovascular health, obesity, child growth

For peer review only - http://bmjopen.bmj.com/site/about/guidelines.xhtml

Article summary: strengths and limitations of this study

1. This is the largest study to date to examine the question of whether childhood BMI and early onset obesity offer cardiometabolic protection to people who are obese as adults.
2. Using non-fasting measures of cholesterol and glucose and pulse pressure, we categorised men in mid life as either metabolically healthy, obese (MHO), metabolically abnormal, obese (MAO), metabolically healthy, normal weight (MHNW) or metabolically abnormal, normal weight (MANW), and examined the relationships of childhood BMI growth and early onset obesity with this phenotype.
3. A limitation of this study is that our measures of cholesterol and glucose were from non-fasting blood samples. There is therefore potential misclassification in our measure of 'metabolically healthy'. We have tried to address this difficulty by using thresholds defined by percentiles of our data, selecting a relatively high cut-off for glucose in order to have reasonable specificity and sensitivity. Given that our estimated prevalence of MHO is within the range seen in previous studies, we feel that this is unlikely to have strongly biased our results.
4. We found no consistent evidence that rapid BMI growth in childhood, or early onset obesity, is associated with a favourable cardiometabolic profile, contradicting previous smaller studies that have examined this question.
5. Our findings imply that the notion of greater severity and metabolic impact of weight gain during adulthood is doubtful and that prevention measures should be implemented in the early childhood years.

For peer review only - http://bmjopen.bmj.com/site/about/guidelines.xhtml

Background:

Recent studies, including reviews[1-3], have drawn attention to the phenotype 'metabolically healthy, but obese' (MHO).[4;5] MHO individuals are defined as those who are obese but have a 'healthy' cardiometabolic profile; precise definitions have varied between studies, but tend to include one or more of insulin resistance, lipids, blood pressure, and markers of inflammation. The prevalence of MHO is estimated to be between 10-40% of obese people (with the remainder classified as metabolically at risk, obese, MAO).[1;6-9] Likewise, non-obese populations can be classified as either 'metabolically healthy, normal weight' (MHNW) or 'metabolically at risk, normal weight' (MANW).

Some studies have shown that MHO is associated with a reduced risk of mortality, cardiovascular disease events, type II diabetes, or a lower carotid intima-media thickness compared with MAO.[10-14] Other studies, however, have demonstrated similar or greater rates of development of hypertension[15-17] and type II diabetes[16;17] in MHO compared with MAO individuals, or high rates of conversion of MHO individuals to an MAO state[18;19], suggesting that MHO is an intermediate step in the development of MAO rather than a static condition.

Several factors have been shown to be positively associated with MHO, e.g. female gender, higher physical activity (though not in all studies[20]), greater fitness[21], longer sleep duration[20], and a favourable psychosocial profile.[1] There is also some evidence that childhood growth is associated with MHO; one small study (N=117 obese children, mean age 10.4 years) found higher birth weight and faster weight gain in infancy was associated with greater levels of circulating insulin and insulin resistance, whereas weight gain after age 4 years was negatively associated with insulin resistance.[22] A further study of 43 postmenopausal women observed that an earlier age at onset of obesity is more common in MHO, with 48% of MHO women reporting an age of obesity onset <20 years compared with 29% of MAO women.[23] These findings are intriguing, given the well-established association between elevated BMI in childhood and greater risk of coronary heart disease in adulthood.[24;25] Research examining the influence of growth in childhood or early-onset obesity and MHO is, however, rare, since few studies have detailed data on both childhood growth and cardiometabolic health in adulthood.

In this paper, we take a cohort of men from Denmark, for whom data are available on childhood growth, early onset obesity, and cardiometabolic risk factors measured in mid-life. We examine the associations of early-onset obesity and trajectories of BMI growth in childhood (from seven to 13

For peer review only - http://bmjopen.bmj.com/site/about/guidelines.xhtml

years) with MHO, MAO, MHNW and MANW.

Materials and Methods:

Participants, exposure measurements and sample definition:

National service is compulsory in Denmark, and before entry into service, draftees attend a physical examination where their fitness to serve is assessed. In the 1970s, a study was initiated to make use of the anthropometric data collected at these examinations. From the 362,200 men examined in the metropolitan area of Copenhagen between 1943-1977, all 1,930 men who were obese (defined as 35% overweight relative to a Scandinavian standard in use at the time, equivalent to a body mass index (BMI) ≥31kg/m^2) and a random sample of 1% of the others (N=3,601) were selected to form a cohort study. Only 3% of men eligible for national service were not examined for medical reasons, with obesity not being a condition that allowed exemption.[26] A further 2% of men were not examined because they had volunteered for service before the age of 18 years. Height was measured without shoes, and weight in only underwear. The mean age at examination was 20 years, range 18-31.

Two re-examinations were undertaken of this cohort, firstly in 1982-1984 (between 4-40 years after the draft board examination, mean age 35 years, range 22-64) and secondly in 1991-1994 (mean age of participants 45.6 years, range 33-75). All of the cohort members who were obese at draft board examination and (for logistical reasons) half of the controls were invited to these clinics, which were attended by 2,075 and 1,703 participants respectively. Further details of this cohort are published elsewhere.[26-30] As part of a separate study[31;32], anthropometric data extracted from school health records between ages 7 and 13 are available for a subset of individuals. Participants gave informed consent, and the protocol was consistent with the principles of the Declaration of Helsinki.

For our main analysis (association between early onset obesity and obesity-metabolic status in mid-life), we included all participants who attended either the first or second follow-up clinic and had their BMI, systolic and diastolic blood pressure (SBP/DBP), cholesterol and glucose measured. We used data from the second follow-up clinic where available, or the first follow-up clinic for participants attending the first but not second assessment. For our secondary analysis of the association between childhood growth trajectories and obesity-metabolic status in mid-life, this sample was further restricted to participants with one or more BMI measure from school health records between 7-13 years.

For peer review only - http://bmjopen.bmj.com/site/about/guidelines.xhtml

Exposure measurements:

Obesity status (BMI ≥31kg/m^2) in early adulthood (at entry into National Service, mean age 20 years) determines our measurement of early-onset obesity.

We used a multilevel model to estimate BMI at age 7 and the linear change in BMI between 7-13 years for all participants with one or more BMI measure. Multilevel models are an appropriate tool for the analysis of longitudinal data, since they allow for the non-independence of repeated measures on the same individual, and can estimate a full trajectory for all participants with one or more measurement under a missing at random assumption.[33;34] The multilevel model was run in MLwiN version 2.26[35] via Stata version 12[36], using the command runmlwin.[37] Full details of the model are provided in online supplementary material. From the multilevel model, we derived predicted BMI at age 7 and predicted linear change in BMI between 7-13 years; these variables were used as exposures in our analyses.

Outcome measurements:

At each follow-up clinic, height was measured without shoes and weight was measured in light clothes and without shoes. SBP and DBP were measured three times with the participant at rest; the mean of the 3 measures was used. Total serum glucose and cholesterol were assessed from non-fasting blood samples drawn from a vein after light stasis. Samples were placed in a box containing ice water, and within two hours centrifuged at 3,000rpm for ten minutes. The plasma was stored at 4°C and analysed the following day. Cholesterol was measured enzymatically using the CHOD-PAP method at the first examination and high-performance CHOD-PAP at the second (Boehringer Mannheim). Glucose was assayed using the hexokinase/G6P-DH assy. Blood samples remained at room temperature for 20-60 minutes, after which the whole blood from the EDTA tube was placed in a plastic tube and frozen at -20°C. To control for treatment effects, a constant of 10mmHg was added to SBP and 5mmHg to DBP for individuals reporting treatment with antihypertensive medication (n=156); these values are consistent with the effects of antihypertensive medications, and this method has been shown to be a reasonable approach for reducing treatment bias and has been used in previous studies.[38-40] Pulse pressure, identified in previous research as an important independent risk factor for cardiovascular disease[41], was defined as SBP-DBP.

For each cardiometabolic risk factor (SBP, DBP, pulse pressure, glucose and cholesterol), a measure of the level of the risk factor given what would be predicted from age and BMI was created by regressing the risk factor on age and BMI at the time of assessment, and standardising the resulting

For peer review only - http://bmjopen.bmj.com/site/about/guidelines.xhtml

residuals to have a mean of zero and variance of one. Thus for these measures a value of zero would mean that the level of the risk factor is what would be predicted given age and BMI, positive values would mean higher than expected levels, and vice versa for negative values.

Various definitions of MHO have been used in previous studies.[1;2] Since the blood samples in our study were non-fasting, standard cut-offs based on fasting samples would be too low. We therefore opted to define cut-offs for cholesterol and glucose based on percentiles of the study-population distribution. For cholesterol, we deemed levels above the 75th centile (6.6mmol/l) to be high. Fluctuations in glucose are greater than those in cholesterol, so a higher threshold is necessary in order to obtain a measure that is reasonably sensitive but also has sufficient specificity. Therefore we defined high glucose levels as above the 95% centile of non-obese participants (8.39mmol/l). In line with previous research[41], we defined high pulse pressure as above 48. 'Metabolically healthy' was defined as having cholesterol, glucose and pulse pressure levels below these thresholds; participants with one or more measurement above these thresholds were deemed 'metabolically at risk'. Individuals were classified according to their obesity status in mid-life (obesity defined as ≥30 kg/m^2) and metabolic status, such that individuals were MHO, MAO, MHNW or MANW.

Other data:

The occupation of participants' fathers was self-reported in questionnaires and coded as manual or non-manual. A binary indicator of participants' smoking behaviour (any versus no smoking) was created from self-reported data.

Statistical analysis:

The associations of early onset obesity and childhood growth with the cardiometabolic risk factors and standardised residuals from regression of cardiometabolic risk factors on age and BMI were assessed using linear regression. The associations of early onset obesity and childhood growth with MHO (compared with MAO) or with MHNW (compared with MANW) were assessed using logistic regression. As a sensitivity analysis, we assessed whether our findings are robust to adjustment for father's social class and participants' smoking status.

Results:

For peer review only - http://bmjopen.bmj.com/site/about/guidelines.xhtml

2,392 participants were included in our analyses; of these, data from the second follow-up assessment were available for 1,691 and from the first assessment for a further 701 (**Table 1**).

Prevalence of obesity and metabolic phenotypes:

Amongst the 2,392 participants, 1,295 were not obese. Of these, 469 (36.2%) were classified as MHNW, and 826 (63.8%) as MANW (**Table 1**). Thus 63.8% of normal weight individuals had one or more metabolic abnormality. Of the 1,097 participants who were obese at follow-up, 297 (27.1%) were MHO.

Compared with MANW individuals, MHNW individuals tended to be younger (mean and standard deviation (SD) for age 44.93 (10.89) and 42.09 (8.40) years respectively) and thinner (mean and SD for BMI 25.55 (2.69) and 24.82 (2.85) years) (**Table 2**). MAO had higher BMI compared with MHO individuals (mean and SD BMI 36.49 (5.10) and 35.79 (4.53) kg/m^2), but there was little difference in age (**Table 2**).

Childhood growth trajectories:

Differences in BMI between participants who were obese and non-obese at draft board examination were already evident by age 7. The mean BMI at age 7 and rate of BMI change between 7-13 years were higher in those who were obese at draft board (**Web Table 1**). The multilevel model had good fit to the data (**Web Table 2** and **Web Figures 1-3**).

Associations of childhood growth and early onset obesity with cardiometabolic health and obesity/metabolic phenotypes in mid-life:

Those with early onset obesity (i.e. those who were obese at draft board examination) had a BMI in mid-life on average 9.65 kg/m^2 (95% CI 9.27 to 10.03) greater than those who were not obese at the draft board examination. Greater BMI at age 7, faster rate of BMI increase between 7-13 years and early onset obesity were all associated with greater SBP, DBP and pulse pressure, and greater glucose levels in mid-life (**Table 3**). These associations tended to increase in magnitude after adjustment for age at the research clinic and year of draft board examination. The associations of childhood growth and early onset obesity with levels of cholesterol were weaker than for the other cardiometabolic risk factors.

There was very little evidence of associations of childhood growth and early onset obesity with standardised scores of the cardiometabolic risk factors that account for age and BMI at the time of

For peer review only

For peer review only - http://bmjopen.bmj.com/site/about/guidelines.xhtml

assessment (**Table 4**). There was no evidence of associations for the standardised scores of SBP or DBP. For standardised scores of glucose, early onset obesity and faster rate of BMI increase between 7-13 years were both associated with higher scores (no association was seen for BMI at 7 years). For example, after adjustment for year at draft board, early onset obesity was associated with a 0.16 SD higher standardised score of glucose (95% CI 0.08 to 0.24), and a 1 kg/m^2 per year increase in the rate of BMI growth between 7-13 years (equivalent to approximately 2 SD of the rate of growth) was associated with a 0.26 SD higher standardised score (95% CI 0.03 to 0.49). Conversely, early onset obesity, greater BMI at age 7 and faster rate of BMI increase between 7-13 years were all associated with lower standardised scores of cholesterol. For example, after adjustment for year of draft board examination, early onset obesity was associated with a -0.19 lower standardised score for cholesterol (95% CI -0.27 to -0.11) and a 1 kg/m^2 per year increase in the rate of BMI growth between 7-13 years was associated with a -0.12 lower standardised score (95% CI -0.35 to 0.10). Thus early onset obesity and faster rates of BMI increase in childhood are associated with a higher glucose level but lower cholesterol level than would be predicted from age and adult BMI.

Among those who were not obese at the follow-up clinic, early onset obesity was associated with slightly lower odds of being MHNW compared with MANW, but the confidence interval was wide and included the null value (odds ratio 0.76, 95% CI 0.54 to 1.07) (**Table 5**). There was no consistent relationship between childhood BMI trajectories and MHNW compared with MANW.

Among those who were obese in mid-life, there was a weak association between early onset obesity and lower odds of being MHO compared with MAO; odds ratio 0.69, 95% CI 0.46 to 1.04. There was no association between BMI at age 7 or BMI increases between 7-13 years and MHO (**Table 5**). Results were unchanged when adjusted for father's social class and participant's smoking (**Web Table 3**).

Discussion:

We found little consistent evidence to support the hypothesis that rapid BMI growth in childhood and early onset obesity are associated with a protective cardiometabolic effect. Early onset obesity and faster rates of BMI increase in childhood were associated with a higher glucose level but lower cholesterol level than would be predicted from age and adult BMI. No associations were seen for SBP or DBP levels or pulse pressure adjusted for concurrent age and BMI. There was also no

For peer review only - http://bmjopen.bmj.com/site/about/guidelines.xhtml

For peer review only

consistent evidence that early onset obesity or rates of BMI change in childhood were associated with being 'metabolically healthy' – early onset obesity was weakly associated with lower odds of being MHO, but no associations were seem for BMI at age 7 or rate of BMI change between 7-13 years. Our findings contrast with a study of 43 postmenopausal women, which observed that an earlier age at onset of obesity is more common in MHO, with 48% of MHO women reporting an age of obesity onset <20 years compared with 29% of MAO women.[23] One small previous study of 117 obese children found that faster weight gain in infancy with greater levels of circulating insulin and reduced insulin resistance, whereas weight gain after age 4 years was associated with higher insulin resistance.[22] The earliest available childhood BMI measures in our cohort are from age 7; thus it is possible that BMI changes at earlier stages could be associated with being metabolically healthy. Our findings are supported by some previous research on this cohort, which has found that the men who were obese at their draft board examination in early adulthood experience a doubling of all-cause mortality risk[42], as well as increased risks of mortality from cardiovascular diseases and a wide range of other specific causes.[43] However, other analyses of this cohort have demonstrated that obese men, maintaining their weight since the draft board examination in early adulthood, had lower risk of impaired glucose tolerance than non-obese men in early adulthood who became similarly obese by age 51.[44]

In this cohort of Danish men, 27.1% of participants who were obese at follow-up were classified as MHO defined by having non-fasting cholesterol <6.6mmol/l, non-fasting glucose <8.39mmol/l and pulse pressure <48mmHg. This is within the range of 10-40% prevalence reported in previous studies.[1;6-9] A large study of over 5000 adults in the National Health and Nutrition Examination Surveys from the USA found a prevalence of MHO of 51.3%.[45] However, this study differed from ours in two important ways; firstly by considering overweight participants rather than obese, and secondly by considering metabolically at risk to be two or more abnormalities, whereas we assumed one or more abnormality conferred risk.

Notably, a very high proportion of non-obese participants were classified as MANW: 63.8%. Analysis of data from a Chinese cohort also found a high prevalence of cardiometabolic abnormalities in non-obese adults[46], leading the authors to conclude that focusing cardiovascular screening only on obese individuals would miss a large proportion of the at risk population. The NHANES data also found that 23.5% of non-overweight participants had at least two metabolic abnormalities.[45] In our study, the mean BMI in the non-obese group was high – 24.82 kg/m^2 in the MHNW group and 25.55 in the MANW group. Thus although not obese, many of these participants were overweight.

For peer review only - http://bmjopen.bmj.com/site/about/guidelines.xhtml

For peer review only

Our analysis included 2,392 individuals, 1,097 of whom were obese, making it larger than most existing studies on MHO.[1;2] We were only able to include a subset of these individuals (N=613) in our analysis of the association between childhood BMI growth and later metabolic health. This is a larger sample size than the former studies on this topic[22;23], but we may still have had limited power to detect an association. Furthermore, some of our participants were quite young at the follow-up examinations, and so associations may emerge in later life as cardiovascular risk develops.

We did not have data on insulin or on lipids other than total cholesterol, and therefore we could not incorporate these into our definitions of MHO, MAO, MHNW and MANW. Our measures of cholesterol and glucose were from non-fasting blood samples. There is therefore potential misclassification in our measure of 'metabolically healthy'. We have tried to address this difficulty by using thresholds defined by percentiles of our data. Glucose levels are likely to fluctuate more in non-fasting samples than cholesterol levels[47], and we therefore selected a relatively high cut-off for glucose in order to have reasonable specificity and sensitivity. Given that our estimated prevalence of MHO is within the range seen in previous studies, we feel that this is unlikely to have strongly biased our results.

We found no consistent evidence that rapid BMI growth in childhood, or early onset obesity, is associated with a favourable cardiometabolic profile, contradicting previous smaller studies that have examined this question. Our findings imply that the notion of greater severity and metabolic impact of weight gain during adulthood is doubtful and that prevention measures should be implemented in the early childhood years.

For peer review only - http://bmjopen.bmj.com/site/about/guidelines.xhtml

Contributions of each author: The study was conceived by LDH and TIAS; the study was designed by LDH with input from TIAS and RW; LDH carried out statistical analyses and wrote the first draft of the manuscript; all authors contributed to critical revisions of the manuscript and approved the final submitted version

Data sharing statement: statistical code can be requested from the corresponding author. For details of data access and collaboration policy, please contact Thokild Sørensen: tias@sund.ku.dk

Funding statement: This work was supported by a UK Medical Research Fellowship to LDH (G1002375); the funders had no role in the design or conduct of the study or the decision to publish.

Competing interests statement: Competing interests: None declared

For peer review only

For peer review only - http://bmjopen.bmj.com/site/about/guidelines.xhtml

Table 1: Sample characteristics, mean (SD) or % characteristics of participants included in analyses

	Participants included in analysis of the association between early-onset obesity and metabolic health in mid-life			Participants included in analysis of the association between childhood growth and metabolic health in mid-life		
	Participants attending second follow-up in adulthood	Participants attending first follow-up in adulthood but not the second follow-up	Combined whole sample	Participants attending second follow-up in adulthood	Participants attending first follow-up in adulthood but not the second follow-up	Combined whole sample
	**N=1,691**	**N=701**	**N=2,392**	**N=420**	**N=193**	**N=613**
Age (years)	45.61 (7.93)	34.81 (8.38)	42.44 (9.45)	48.01 (6.82)	36.34 (6.90)	44.34 (8.73)
Number (%) obese in early adulthood*	784 (46.4%)	346 (49.4%)	1,130 (47.2%)	189 (45.0%)	95 (49.2%)	284 (46.3%)
Body mass index (kg/m^2)	30.57 (6.69)	29.76 (6.87)	30.34 (6.75)	30.52 (6.79)	31.00 (7.74)	30.67 (7.10)
Systolic blood pressure (mmHg)	142.37 (18.12)	138.50 (16.18)	141.89 (18.32)	143.64 (18.75)	138.08 (16.33)	141.89 (18.19)
Diastolic blood pressure (mmHg)	92.81 (11.41)	86.45 (12.25)	91.27 (12.31)	92.29 (10.81)	89.27 (11.70)	91.34 (11.18)
Pulse pressure (mmHg)	49.96 (13.26)	52.22 (12.38)	50.62 (13.04)	51.36 (13.73)	48.80 (11.91)	50.55 (13.23)
Glucose (mmol/l)	6.48 (2.95)	6.57 (2.42)	6.51 (2.80)	6.56 (3.14)	6.65 (2.52)	6.59 (2.96)
Cholesterol (mmol/l)	6.10 (1.28)	5.25 (1.22)	5.85 (1.32)	6.11 (1.20)	5.34 (1.24)	6.86 (1.26)
*Number (%) with metabolic phenotypes ***						
NON-OBESE AT MID-LIFE FOLLOW-UP	*N=903*	*N=392*	*N=1,295*	*N=235*	*N=102*	*N=337*
Metabolically healthy	328 (36.3%)	141 (36.0%)	469 (36.2%)	81 (34.5%)	49 (48.0%)	130 (38.6%)
Metabolically at risk	575 (63.7%)	251 (64.0%)	826 (63.8%)	154 (65.5%)	53 (52.0%)	207 (61.4%)
OBESE AT MID-LIFE FOLLOW-UP	*N=788*	*N=309*	*N=1,097*	*N=185*	*N=91*	*N=276*
Metabolically healthy, obese	221 (28.0%)	76 (24.6%)	297 (27.1%)	48 (25.9%)	28 (30.8%)	76 (27.5%)
Metabolically at risk, obese	567 (72.0%)	233 (75.4%)	800 (72.9%)	137 (74.1%)	63 (69.2%)	200 (72.5%)

*Defined as 35% overweight relative to a Scandinavian standard in use at the time, equivalent to a body mass index (BMI) ≥31kg/m^2

**Metabolically healthy is defined by having pulse pressure (systolic – diastolic blood pressure) <48mmHg, glucose <8.39mmol/l, and cholesterol <6.6mmol/l; i.e. any one or more metabolic abnormality confers 'metabolically at-risk' status. Obesity at the mid-life follow-up is defined by BMI≥30kg/m^2

For peer review only - http://bmjopen.bmj.com/site/about/guidelines.xhtml

Table 2: Characteristics of participants within each metabolic and obesity category

	Metabolically healthy, normal weight (MHNW)*	Metabolically at risk, normal weight (MANW)*	Metabolically healthy, obese (MHO)*	Metabolically at risk, obese (MAO)*
Mean (SD)				
	N=469	N=826	N=297	N=800
Age (years)	42.09 (8.40)	44.93 (10.89)	40.15 (7.33)	40.93 (8.53)
BMI (kg/m^2)	24.82 (2.85)	25.55 (2.69)	35.79 (4.53)	36.49 (5.10)
Systolic blood pressure (mmHg)	126.60 (10.37)	142.97 (16.98)	134.70 (10.74)	152.41 (18.22)
Diastolic blood pressure (mmHg)	86.85 (9.21)	88.00 (12.34)	95.21 (9.77)	95.77 (12.76)
Pulse pressure (mmHg)	39.74 (5.23)	54.97 (11.97)	39.49 (5.67)	56.64 (12.73)
Cholesterol (mmol/l)	5.26 (0.87)	6.06 (1.40)	5.39 (0.82)	6.15 (1.45)
Glucose (mmol/l)	5.62 (0.87)	6.29 (2.30)	5.76 (0.74)	7.53 (3.95)
Number (%) that were obese in early adulthood**	61 (13.0%)	118 (14.3%)	253 (85.2%)	698 (87.3%)

* Metabolically healthy is defined by having pulse pressure (systolic – diastolic blood pressure) <48mmHg, glucose <8.39mmol/l, and cholesterol <6.6mmol/l; i.e. any one or more metabolic abnormality confers 'metabolically at-risk' status. Obesity in mid-life is defined by BMI≥30kg/m^2

**Obesity in early adulthood is defined as 35% overweight relative to a Scandinavian standard in use at the time, equivalent to a body mass index (BMI) ≥31kg/m^2

For peer review only

For peer review only - http://bmjopen.bmj.com/site/about/guidelines.xhtml

Table 3: Linear regression coefficients (with 95% confidence intervals) for the association of early onset obesity and childhood BMI trajectories with cardiovascular risk factors measured in mid-life.

Model*	Exposure	Outcome					
		BMI (kg/m^2)	Systolic blood pressure (mmHg)	Diastolic blood pressure (mmHg)	Pulse pressure (mmHg)	Glucose (mmol/l)	Cholesterol (mmol/l)
Full sample, N=2,329							
1	Early onset obesity**	9.65 (9.27 to 10.03) p<0.001	7.70 (6.27 to 9.14) p<0.001	5.31 (4.35 to 6.28) p<0.001	2.39 (1.35 to 3.44) p<0.001	1.14 (0.92 to 1.36) p<0.001	-0.14 (-0.25 to -0.04) p=0.01
2		9.76 (9.36 to 10.16) p<0.001	10.28 (8.82 to 11.74) p<0.001	6.02 (5.01 to 7.02) p<0.001	4.27 (3.21 to 5.33) p<0.001	1.30 (1.07 to 1.53) p<0.001	-0.07 (-0.18 to 0.04) p=0.19
Subsample with childhood growth data, N=613							
1	BMI at 7 years (kg/m^2)	1.81 (1.60 to 2.02) p<0.001	1.37 (0.72 to 2.02) p<0.001	0.72 (0.32 to 1.12) p<0.001	0.65 (0.17 to 1.13) p=0.01	0.12 (0.02 to 0.23) p=0.03	-0.04 (-0.09 to 0.00) p=0.08
2		1.75 (1.53 to 1.97) p<0.001	1.82 (1.17 to 2.47) p<0.001	0.85 (0.44 to 1.26) p<0.001	0.97 (0.49 to 1.45) p<0.001	0.13 (0.02 to 0.24) p=0.02	-0.02 (-0.06 to 0.03) p=0.50
1	BMI change 7-13 years (kg/m^2 per year)	10.95 (10.01 to 11.89) p<0.001	8.47 (5.26 to 11.67) p<0.001	4.53 (2.55 to 6.51) p<0.001	3.93 (1.57 to 6.30) p=0.001	1.31 (0.78 to 1.83) p<0.001	-0.20 (-0.42 to 0.03) p=0.09
2		8.75 (7.59 to 9.91) p<0.001	9.69 (5.76 to 13.63) p<0.001	4.76 (2.27 to 7.25) p<0.001	4.93 (2.00 to 7.87) p=0.001	1.58 (0.91 to 2.24) p<0.001	0.05 (-0.22 to 0.33) p=0.71

*Model details: Model 1 is unadjusted; Model 2 is adjusted for age at outcome assessment and year of draft board examination, and the coefficient for change in BMI between 7-11 years is additionally adjusted for BMI at 7 years as predicted by the multilevel model

**Defined as 35% overweight relative to a Scandinavian standard in use at the time, equivalent to a body mass index (BMI) ≥31kg/m^2 in early adulthood, mean age 20 years

For peer review only - http://bmjopen.bmj.com/site/about/guidelines.xhtml

Table 4: Linear regression coefficients (with 95% confidence intervals) for the association of early onset obesity and childhood BMI trajectories with levels of cardiovascular risk factors in mid-life adjusted for age and BMI in mid-life.

Model*	Exposure	Outcomes** (standard deviations, given age and concurrent BMI)				
		Systolic blood pressure	Diastolic blood pressure	Pulse pressure	Glucose	Cholesterol
Full sample, N=2,329						
1	Early onset obesity***	0.02 (-0.06 to 0.10) p=0.62	-0.01 (-0.09 to 0.07) p=0.78	0.04 (-0.04 to 0.12) p=0.38	0.13 (0.05 to 0.21) p=0.002	-0.13 (-0.21 to -0.05) p=0.002
2		0.05 (-0.04 to 0.13) p=0.28	-0.06 (-0.14 to 0.03) p=0.18	0.11 (0.03 to 0.19) p=0.01	0.16 (0.08 to 0.24) p<0.001	-0.19 (-0.27 to -0.11) p<0.001
Subsample with childhood growth data, N=613						
1	BMI at 7 years (kg/m^2)	0.00 (-0.04 to 0.04) p=0.97	-0.01 (-0.05 to 0.03) p=0.57	0.01 (-0.03 to 0.05) p=0.61	-0.01 (-0.05 to 0.02) p=0.42	-0.03 (-0.07 to 0.00) p=0.07
2		0.00 (-0.03 to 0.04) p=0.89	-0.01 (-0.05 to 0.02) p=0.47	0.01 (-0.02 to 0.05) p=0.49	-0.01 (-0.05 to 0.02) p=0.50	-0.04 (-0.08 to -0.01) p=0.02
1	BMI change 7-13 years (kg/m^2 per year)	0.03 (-0.15 to 0.21) p=0.75	-0.04 (-0.22 to 0.14) p=0.69	0.07 (-0.11 to 0.25) p=0.46	0.11 (-0.07 to 0.29) p=0.24	-0.15 (-0.33 to 0.03) p=0.10
2		0.05 (-0.18 to 0.28) p=0.65	-0.02 (-0.25 to 0.20) p=0.83	0.07 (-0.16 to 0.31) p=0.52	0.26 (0.03 to 0.49) p=0.03	-0.12 (-0.35 to 0.10) p=0.28

The outcome in these analyses is a measure of the level of the cardiovascular risk factor (SBP, DBP, pulse pressure, glucose and cholesterol) given what would be predicted from age and BMI; this was created by regressing the risk factor on age and BMI at the time of assessment, and standardising the resulting residuals to have a mean of zero and variance of one. Thus for these measures a value of zero would mean that the level of the risk factor is what would be predicted given age and BMI, positive values would mean higher than expected levels, and vice versa for negative values.

*Model details: Model 1 is unadjusted; Model 2 is adjusted for year of draft board examination, in model 2 the coefficient for change in BMI between 7-11 years is additionally adjusted for BMI at 7 years as predicted by the multilevel model

**Cardiovascular risk factors were regressed on concurrent BMI and age, and standardised residuals (mean of zero, variance of one) from those regressions are used as the outcomes in these analyses. Thus a positive value of these outcomes means that an individual has a higher level of that outcome than would be expected given their age and BMI, and a negative value means the opposite, lower level of the outcome given their age and BMI

***Defined as 35% overweight relative to a Scandinavian standard in use at the time, equivalent to a body mass index (BMI) ≥31kg/m^2 in early adulthood, mean age 20 years

For peer review only

For peer review only - http://bmjopen.bmj.com/site/about/guidelines.xhtml

Table 5: Odds ratios (with 95% confidence intervals) for the association of obese status in early adulthood and growth in early childhood with metabolic status in mid-life; stratified by mid-life obesity status

Model*	**Metabolically healthy, normal weight****	**Metabolically at risk, normal weight****	**Metabolically healthy, obese****	**Metabolically at risk, obese****
Full sample, N=2,329				
	N=469	*N=826*	*N=297*	*N=800*
**Early onset obesity*****				
1	0.90 (0.64 to 1.25) p=0.52	1 (ref)	0.84 (0.57 to 1.23) p=0.37	1 (ref)
2	0.76 (0.54 to 1.07) p=0.12	1 (ref)	0.69 (0.46 to 1.04) p=0.08	1 (ref)
Subsample with childhood growth data, N=613				
	N=130	*N=207*	*N=76*	*N=200*
**BMI at 7 (kg/m^2)**				
1	0.97 (0.85 to 1.10) p=0.60	1 (ref)	0.99 (0.88 to 1.13) p=0.92	1 (ref)
2	0.93 (0.81 to 1.07) p=0.32	1 (ref)	0.97 (0.85 to 1.10) p=0.65	1 (ref)
**BMI change 7-13 (kg/m^2 per year)**				
1	0.85 (0.41 to 1.70) p=0.62	1 (ref)	1.50 (0.73 to 3.05) p=0.27	1 (ref)
2	0.66 (0.25 to 1.72) p=0.39	1 (ref)	1.40 (0.61 to 3.48) p=0.38	1 (ref)

Coefficients are odds ratios (95% confidence intervals) comparing obese in early adulthood with non-obese in early adulthood, or a one unit increase in BMI at age 7 or the linear rate of change in BMI between 7 and 13 years.

*Model details: Model 1 is unadjusted; Model 2 is adjusted for age at outcome assessment and year of draft board examination. In model 2 the coefficient for change in BMI between 7-13 years is additionally adjusted for BMI at 7 years as predicted by the multilevel model

** Metabolically healthy is defined by having pulse pressure (systolic – diastolic blood pressure) <48mmHg, glucose <8.39mmol/l, and cholesterol <6.6mmol/l; i.e. any one or more metabolic abnormality confers 'metabolically at-risk' status. Obesity in mid-life is defined by BMI≥30kg/m^2

***Defined as 35% overweight relative to a Scandinavian standard in use at the time, equivalent to a body mass index (BMI) ≥31kg/m^2 in early adulthood, mean age 20 years

For peer review only

For peer review only - http://bmjopen.bmj.com/site/about/guidelines.xhtml

Reference List

(1) Primeau V, Coderre L, Karelis AD, Brochu M, Lavoie ME, Messier V et al. Characterizing the profile of obese patients who are metabolically healthy. *Int J Obes* 2011; 35(7):971-981.

(2) Denis GV, Obin MS. 'Metabolically healthy obesity': Origins and implications. *Mol Aspects Med* 2012.

(3) Ahima RS, Lazar MA. The health risk of obesity - better metrics imperative. *Science* 2013; 341:856.

(4) Sorensen TI, Virtue S, Vidal-Puig A. Obesity as a clinical and public health problem: is there a need for a new definition based on lipotoxicity effects? *Biochim Biophys Acta* 2010; 1801(3):400-404.

(5) Sorensen TI. Obesity defined as excess storage of inert triglycerides--do we need a paradigm shift? *Obes Facts* 2011; 4(2):91-94.

(6) Karelis AD, Brochu M, Rabasa-Lhoret R. Can we identify metabolically healthy but obese individuals (MHO)? *Diabetes Metab* 2004; 30(6):569-572.

(7) Karelis AD, Brochu M, Rabasa-Lhoret R, Garrel D, Poehlman ET. Clinical markers for the identification of metabolically healthy but obese individuals. *Diabetes Obes Metab* 2004; 6(6):456-457.

(8) Karelis AD, St-Pierre DH, Conus F, Rabasa-Lhoret R, Poehlman ET. Metabolic and body composition factors in subgroups of obesity: what do we know? *J Clin Endocrinol Metab* 2004; 89(6):2569-2575.

(9) Karelis AD. Metabolically healthy but obese individuals. *Lancet* 2008; 372(9646):1281-1283.

(10) Meigs JB, Wilson PW, Fox CS, Vasan RS, Nathan DM, Sullivan LM et al. Body mass index, metabolic syndrome, and risk of type 2 diabetes or cardiovascular disease. *J Clin Endocrinol Metab* 2006; 91(8):2906-2912.

(11) Durward CM, Hartman TJ, Nickols-Richardson SM. All-Cause Mortality Risk of Metabolically Healthy Obese Individuals in NHANES III. *J Obes* 2012; 2012:460321.

(12) Hamer M, Stamatakis E. Metabolically healthy obesity and risk of all-cause and cardiovascular disease mortality. *J Clin Endocrinol Metab* 2012; 97(7):2482-2488.

(13) Calori G, Lattuada G, Piemonti L, Garancini MP, Ragogna F, Villa M et al. Prevalence, metabolic features, and prognosis of metabolically healthy obese Italian individuals: the Cremona Study. *Diabetes Care* 2011; 34(1):210-215.

(14) Li S, Chen W, Srinivasan SR, Xu J, Berenson GS. Relation of childhood obesity/cardiometabolic phenotypes to adult cardiometabolic profile: the Bogalusa Heart Study. *Am J Epidemiol* 2012; 176 Suppl 7:S142-S149.

For peer review only

(15) Lee SK, Kim SH, Cho GY, Baik I, Lim HE, Park CG et al. Obesity phenotype and incident hypertension: a prospective community-based cohort study. *J Hypertens* 2013; 31(1):145-151.

(16) Hwang LC, Bai CH, Sun CA, Chen CJ. Prevalence of metabolically healthy obesity and its impacts on incidences of hypertension, diabetes and the metabolic syndrome in Taiwan. *Asia Pac J Clin Nutr* 2012; 21(2):227-233.

(17) Chang Y, Ryu S, Suh BS, Yun KE, Kim CW, Cho SI. Impact of BMI on the incidence of metabolic abnormalities in metabolically healthy men. *Int J Obes (Lond)* 2012; 36(9):1187-1194.

(18) Appleton SL, Seaborn CJ, Visvanathan R, Hill CL, Gill TK, Taylor AW et al. Diabetes and Cardiovascular Disease Outcomes in the Metabolically Healthy Obese Phenotype: A cohort study. *Diabetes Care* 2013.

(19) Bradshaw PT, Monda KL, Stevens J. Metabolic syndrome in healthy obese, overweight, and normal weight individuals: The atherosclerosis risk in communities study. *Obesity (Silver Spring)* 2013; 21(1):203-209.

(20) Hankinson AL, Daviglus ML, Van HL, Chan Q, Brown I, Holmes E et al. Diet composition and activity level of at risk and metabolically healthy obese American adults. *Obesity (Silver Spring)* 2012.

(21) Ortega FB, Lee DC, Katzmarzyk PT, Ruiz JR, Sui X, Church TS et al. The intriguing metabolically healthy but obese phenotype: cardiovascular prognosis and role of fitness. *Eur Heart J* 2013; 34(5):389-397.

(22) Bouhours-Nouet N, Dufresne S, de Casson FB, Mathieu E, Douay O, Gatelais F et al. High birth weight and early postnatal weight gain protect obese children and adolescents from truncal adiposity and insulin resistance: metabolically healthy but obese subjects? *Diabetes Care* 2008; 31(5):1031-1036.

(23) Brochu M, Tchernof A, Dionne IJ, Sites CK, Eltabbakh GH, Sims EA et al. What are the physical characteristics associated with a normal metabolic profile despite a high level of obesity in postmenopausal women? *J Clin Endocrinol Metab* 2001; 86(3):1020-1025.

(24) Baker JL, Olsen LW, Sorensen TI. Childhood body-mass index and the risk of coronary heart disease in adulthood. *New England Journal of Medicine* 2007; 357:2329-2337.

(25) Owen CG, Whincup PH, Orfei L, Chou Q-A, Rudnicka AR, Wathern AK et al. Is body mass index before middle age related to coronary heart disease risk in later life? Evidence from observational studies. *International Journal of Obesity* 2009; 33:866-877.

(26) Sorensen TI, Sonne-Holm S. Mortality in extremely overweight young men. *J Chronic Dis* 1977; 30(6):359-367.

(27) Sonne-Holm S, Sorensen TI, Christensen U. Risk of early death in extremely overweight young men. *Br Med J (Clin Res Ed)* 1983; 287(6395):795-797.

(28) Sonne-Holm S, Sorensen TI, Jensen G, Schnohr P. Long-term changes of body weight in adult obese and non-obese men. *Int J Obes* 1990; 14(4):319-326.

For peer review only - http://bmjopen.bmj.com/site/about/guidelines.xhtml

(29) Sonne-Holm S, Sorensen TI, Jensen G, Schnohr P. Independent effects of weight change and attained body weight on prevalence of arterial hypertension in obese and non-obese men. *BMJ* 1989; 299(6702):767-770.

(30) Sorensen TI, Sonne-Holm S. Risk in childhood of development of severe adult obesity: retrospective, population-based case-cohort study. *Am J Epidemiol* 1988; 127(1):104-113.

(31) Baker JL, Sorensen TI. The Copenhagen School Health Records Register. *Scand J Public Health* 2011; 39(7 Suppl):87-90.

(32) Baker JL, Sorensen TI. Obesity research based on the Copenhagen School Health Records Register. *Scand J Public Health* 2011; 39(7 Suppl):196-200.

(33) Goldstein H. Multilevel statistical models. 2nd edition ed. London: Edward Arnold; 1995.

(34) Tilling K, Davies NM, Nicoli E, Ben-Shlomo Y, Kramer MS, Patel R et al. Associations of growth trajectories in infancy and early childhood with later childhood outcomes. *Am J Clin Nutr* 2011.

(35) MLwiN Version 2.26. Centre for Multilevel Modelling, University of Bristol [2012.

(36) Stata 12.0 [Texas: StataCorp; 2011.

(37) runmlwin: Stata module for fitting multilevel models in the MLwiN software. Centre for Multilevel Modelling, University of Bristol [2011.

(38) Wills AK, Lawlor DA, Matthews FE, Sayer AA, Bakra E, Ben-Shlomo Y et al. Life course trajectories of systolic blood pressure using longitudinal data from eight UK cohorts. *PLoS Med* 2011; 8(6):e1000440.

(39) Tobin MD, Sheehan NA, Scurrah KJ, Burton PR. Adjusting for treatment effects in studies of quantitative traits: antihypertensive therapy and systolic blood pressure. *Stat Med* 2005; 24(19):2911-2935.

(40) Wu J, Kraja AT, Oberman A, Lewis CE, Ellison RC, Arnett DK et al. A summary of the effects of antihypertensive medications on measured blood pressure. *Am J Hypertens* 2005; 18(7):935-942.

(41) Fang J, Madhaven S, Alderman MH. Pulse pressure: a predictor of cardiovascular mortality among young normotensive subjects. *Blood Pressure* 2000; 9:260-266.

(42) Zimmermann E, Holst C, Sorensen TI. Lifelong doubling of mortality in men entering adult life as obese. *Int J Obes (Lond)* 2011; 35(9):1193-1199.

(43) Zimmermann E, Holst C, Sorensen TI. Morbidity, including fatal morbidity, throughout life in men entering adult life as obese. *PLoS One* 2011; 6(4):e18546.

(44) Black E, Holst C, Astrup A, Toubro S, Echwald S, Pedersen O et al. Long-term influences of body-weight changes, independent of the attained weight, on risk of impaired glucose tolerance and Type 2 diabetes. *Diabet Med* 2005; 22(9):1199-1205.

(45) Wildman R, Muntner P, Reynolds K, McGinn AP, Rajpathak K, Wyile-Rosett J et al. The obese without cardiometabolic risk factor clustering and the normal weight with cardiometabolic

For peer review only - http://bmjopen.bmj.com/site/about/guidelines.xhtml

risk factor clustering: prevalence and correlates of 2 phenotypes among the US population (NHANES 1999-2004) . *Arch Intern Med* 2008; 168:1617-1624.

(46) Gordon-Larsen P, Adair LS, Meigs JB, Mayer-Davis E, Herring A, Yan SK et al. Discordant risk: Overweight and cardiometabolic risk in chinese adults. *Obesity (Silver Spring)* 2013; 21(1):E166-E174.

(47) Statland B, Winkel P, Bokelund H. Factors contributing to intra-individual variation of serum constituents: 2. Effects of exercise and diet on variation of serum constituents in healthy subjects. *Clinical Chemistry* 1973; 19:1380-1383.

For peer review only

For peer review only - http://bmjopen.bmj.com/site/about/guidelines.xhtml

Online-only supplementary material

Childhood growth and early-onset obesity in relation to 'metabolically healthy, but obese'

Laura D Howe*[1], Esther Zimmermann[2], Ram Weiss[3], Thorkild IA Sørensen[2,4]

1 – MRC Integrative Epidemiology Unit at the University of Bristol, School of Social and Community Medicine, University of Bristol, Bristol, UK
2 – Institute of Preventive Medicine, Frederiksberg and Bispebjerg Hospitals, The Capital Region, Copenhagen, Denmark
3 – Braun School of Public Health, The Hebrew University of Jerusalem, Israel
4 - Novo Nordisk Center for Basic Metabolic Research, Faculty of Health and Medical Sciences, University of Copenhagen, Denmark

For peer review only

Details of multilevel model used to estimate childhood BMI trajectories

Differences in size between the participants identified as obese at the draft board examination and the random 1% sample of all individuals were already evident at age seven. A separate intercept and slope for participants identified as obese at the draft board examination was included in the multilevel models in order to model these differences. A linear slope was found to adequately describe BMI changes between seven and 13 years. Therefore two variables summarise each individual's BMI trajectory: an intercept describing their BMI at age seven, and a slope describing their linear change in BMI between seven and 13 years, as defined by the following equation:

$$BMI_{ij} = (\beta0+u_{0j}+e_{0ij}) + (\beta1+u_{1j})(Age_{ij}) + \beta2(Case_j) + \beta3(Case*Age_j) + e_{ij}(Age_{ij})$$

Where, for individual j at measurement occasion i:

β0=mean estimate of BMI (kg/m^2) at age 7, β1=mean estimates of rate of BMI change between 7-13 years in kg/m^2 per year, β2=mean difference in BMI at age 7 comparing cases (those who were obese in early adulthood, at draft board examination) with non-cases (those not obese at draft board examination), β3=mean difference in rate of BMI change between 7-13 years comparing cases with non-cases, u=individual level random effects, e=error (occasion level random effects)

From the multilevel model, we derived predicted BMI at age 7 and predicted linear change in BMI between 7-13 years (population average intercept and slope, plus the coefficients for mean difference in intercept or slope for those obese at draft board examination if applicable, plus the individual-level residuals for intercept or slope).

For peer review only

Web table 1: Mean (SD) rate of change in BMI across childhood

	Overall	Obese in early adulthood*	Non-obese in early adulthood	P value for difference between obese and non-obese in early adulthood
	N=613	N=329	N=284	
BMI at age 7 (kg/m^2)	17.00 (0.44)	18.50 (2.12)	15.70 (1.18)	<0.001
Rate of BMI growth 7-13yrs (kg/m^2/year)	0.73 (0.44)	1.12 (0.31)	0.40 (0.19)	<0.001

*Defined as 35% overweight relative to a Scandinavian standard in use at the time, equivalent to a body mass index (BMI) ≥31kg/m^2 in early adulthood, mean age 20 years

For peer review only

Web table 2: Model fit for childhood BMI trajectories.

Age	Total number of individuals measured (number who were obese in early adulthood)	Men who were not obese in early adulthood*				Men who were obese in early adulthood			
		Mean (SD) observed BMI	Mean (SD) predicted BMI	Mean difference between observed and predicted measures	95% reference range for the differences between observed and predicted measurements	Mean (SD) observed BMI	Mean (SD) predicted BMI	Mean difference between observed and predicted measures	95% reference range for the differences between observed and predicted measurements
**7 years**	557 (265)	15.71 (1.25)	15.67 (1.21)	+0.04	-0.61 to 0.69	18.50 (2.26)	18.50 (2.11)	+0.004	-1.37 to 1.38
**8 years**	540 (259)	16.18 (1.38)	16.13 (1.26)	+0.04	-0.72 to 0.80	19.71 (2.42)	19.61 (2.15)	+0.10	-1.28 to 1.48
**9 years**	526 (249)	16.54 (1.53)	16.59 (1.38)	-0.04	-0.85 to 0.76	20.65 (2.61)	20.77 (2.31)	-0.12	-1.90 to 1.67
**10 years**	543 (254)	16.96 (1.68)	17.05 (1.55)	-0.09	-1.07 to 0.90	21.80 (2.88)	21.83 (2.43)	-0.03	-2.10 to 2.04
**11 years**	543 (255)	17.40 (1.86)	17.49 (1.69)	-0.09	-1.08 to 0.90	22.93 (2.94)	22.99 (2.58)	-0.07	-2.04 to 1.90
**12 years**	545 (261)	17.93 (1.97)	17.95 (1.83)	-0.02	-0.96 to 0.93	24.20 (3.09)	24.18 (2.80)	+0.02	-2.07 to 2.12
**13 years**	546 (259)	18.61 (2.19)	18.40 (2.01)	+0.21	-0.97 to 1.40	25.31 (3.13)	25.20 (2.97)	+0.11	-2.54 to 2.77

613 participants had at least one or more measure of BMI between 7 and 13 years.

*Defined as 35% overweight relative to a Scandinavian standard in use at the time, equivalent to a body mass index (BMI) ≥31kg/m^2 in early adulthood, mean age 20 years

For peer review only

Web Table 3: Association of obese status in early adulthood and growth in early childhood with metabolic status in mid-life; stratified by mid-life obesity status with adjustment for confounders

Coefficients are odds ratios (95% confidence intervals) comparing obese in early adulthood with non-obese in early adulthood, or a one unit increase in BMI at age 7 or the linear rate of change in BMI between 7 and 13 years.

Model*	Metabolically healthy, normal weight**	Metabolically at risk, normal weight**	Metabolically healthy, obese**	Metabolically at risk, obese**
**Early onset obesity*** **				
3	N=115<br>0.95 (0.47 to 1.94)<br>p=0.90	N=190<br>1 (ref)	N=64<br>0.92 (0.36 to 2.39)<br>p=0.87	N=187<br>1 (ref)
**BMI at 7 (kg/m^2)**				
3	N=116<br>0.95 (0.83 to 1.10)<br>p=0.51	N=188<br>1 (ref)	N=63<br>1.02 (0.89 to 1.17)<br>p=0.81	N=181<br>1 (ref)
**BMI change 7-13 (kg/m^2 per year)**				
3	N=116<br>0.66 (0.23 to 1.91)<br>p=0.44	N=188<br>1 (ref)	N=63<br>1.46 (0.61 to 3.48)<br>p=0.39	N=181<br>1 (ref)

*Model details: Model 3 is adjusted for age at outcome assessment, year of draft board examination, father's social class and current smoking. The coefficient for change in BMI between 7-13 years is additionally adjusted for BMI at 7 years as predicted by the multilevel model

** Metabolically healthy is defined by having pulse pressure (systolic – diastolic blood pressure) <48mmHg, glucose <8.39mmol/l, and cholesterol <6.6mmol/l; i.e. any one or more metabolic abnormality confers 'metabolically at-risk' status. Obesity in mid-life is defined by BMI≥30kg/m^2

***Defined as 35% overweight relative to a Scandinavian standard in use at the time, equivalent to a body mass index (BMI) ≥31kg/m^2 in early adulthood, mean age 20 years

For peer review only

Web Figure 1: Normal plot of individual level residuals for BMI at age 7 from the multilevel model of childhood BMI trajectories

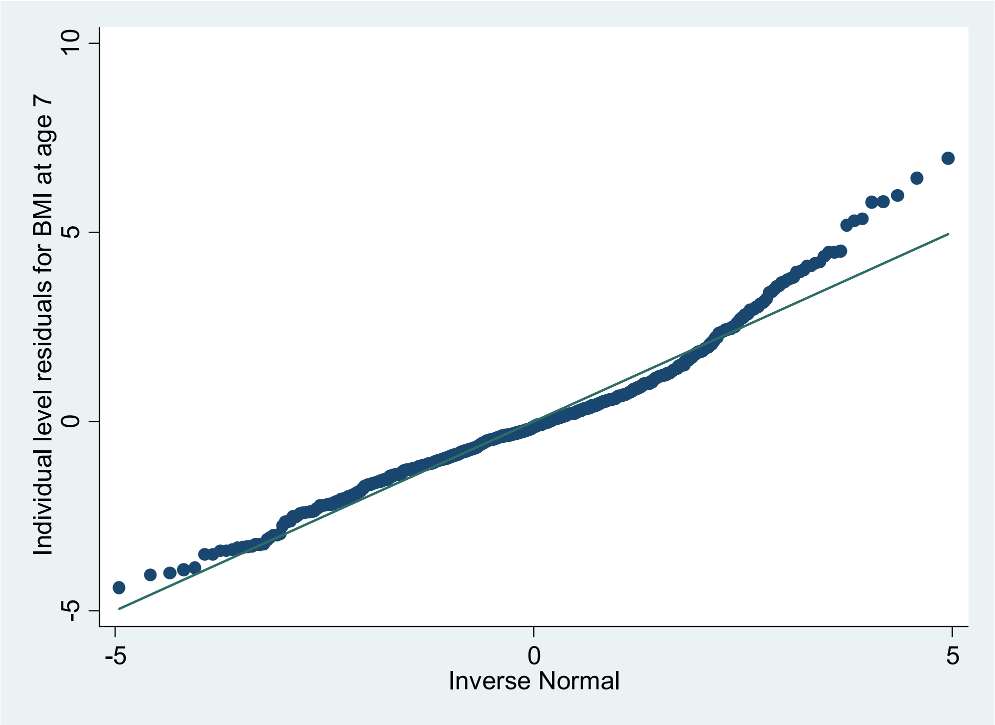

For peer review only

Web Figure 2: Normal plot of individual level residuals for BMI change between 7-13 years from the multilevel model of childhood BMI trajectories

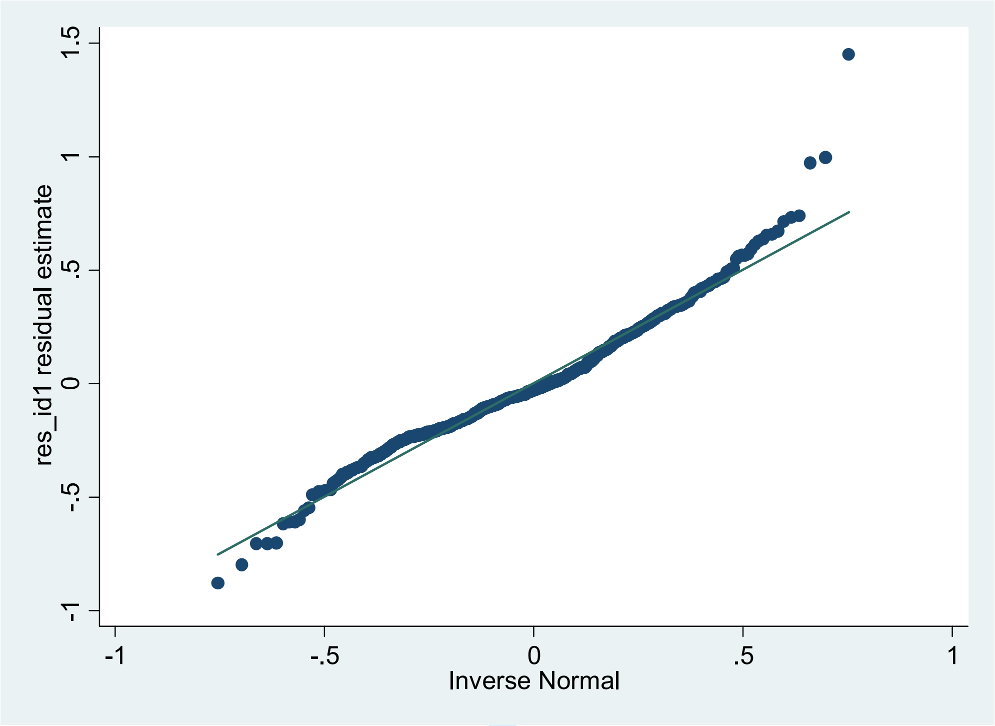

For peer review only

Web Figure 3: Normal plot of occasion level residuals from the multilevel model of childhood BMI trajectories

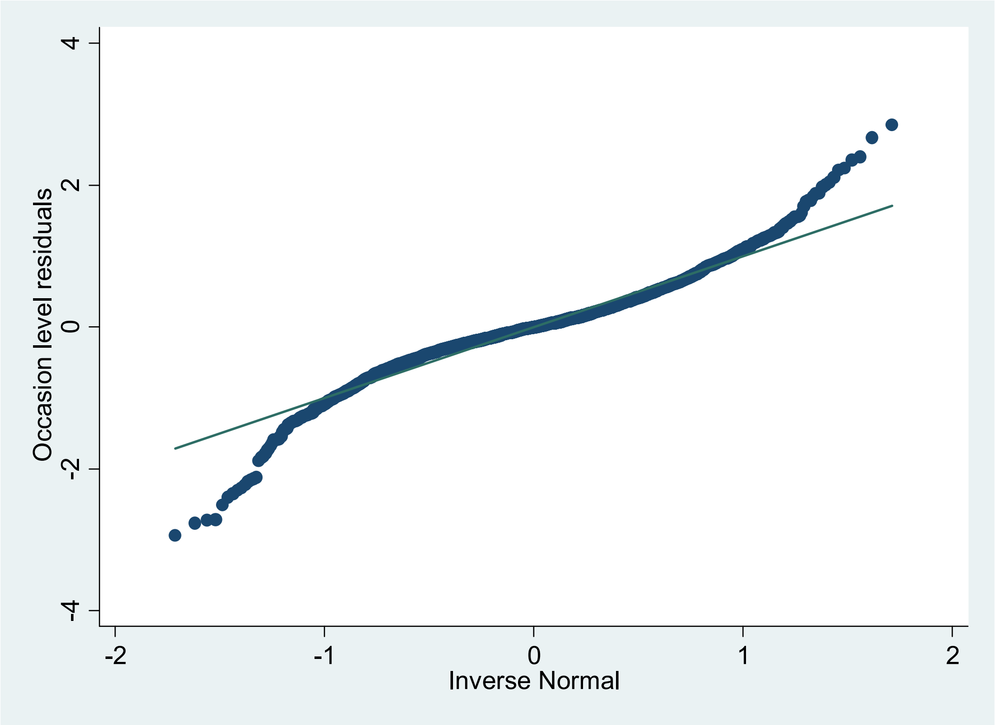

For peer review only

For peer review only

STROBE 2007 (v4) Statement—Checklist of items that should be included in reports of *cohort studies*

Section/Topic	Item #	Recommendation	Reported on page #
**Title and abstract**	1	(*a*) Indicate the study's design with a commonly used term in the title or the abstract	1
		(*b*) Provide in the abstract an informative and balanced summary of what was done and what was found	2
**Introduction**			
Background/rationale	2	Explain the scientific background and rationale for the investigation being reported	4
Objectives	3	State specific objectives, including any prespecified hypotheses	4, 5
**Methods**			
Study design	4	Present key elements of study design early in the paper	5
Setting	5	Describe the setting, locations, and relevant dates, including periods of recruitment, exposure, follow-up, and data collection	5
Participants	6	(*a*) Give the eligibility criteria, and the sources and methods of selection of participants. Describe methods of follow-up	5, 6
		(*b*) For matched studies, give matching criteria and number of exposed and unexposed	n/a
Variables	7	Clearly define all outcomes, exposures, predictors, potential confounders, and effect modifiers. Give diagnostic criteria, if applicable	5, 6
Data sources/ measurement	8*	For each variable of interest, give sources of data and details of methods of assessment (measurement). Describe comparability of assessment methods if there is more than one group	5, 6
Bias	9	Describe any efforts to address potential sources of bias	7
Study size	10	Explain how the study size was arrived at	5
Quantitative variables	11	Explain how quantitative variables were handled in the analyses. If applicable, describe which groupings were chosen and why	5, 6, 7
Statistical methods	12	(*a*) Describe all statistical methods, including those used to control for confounding	7
		(*b*) Describe any methods used to examine subgroups and interactions	7
		(*c*) Explain how missing data were addressed	7
		(*d*) If applicable, explain how loss to follow-up was addressed	7
		(*e*) Describe any sensitivity analyses	7
**Results**			

For peer review only

Participants	13*	(a) Report numbers of individuals at each stage of study—eg numbers potentially eligible, examined for eligibility, confirmed eligible, included in the study, completing follow-up, and analysed	5, 8
		(b) Give reasons for non-participation at each stage	5, 8
		(c) Consider use of a flow diagram	n/a
Descriptive data	14*	(a) Give characteristics of study participants (eg demographic, clinical, social) and information on exposures and potential confounders	8
		(b) Indicate number of participants with missing data for each variable of interest	5, 8
		(c) Summarise follow-up time (eg, average and total amount)	5
Outcome data	15*	Report numbers of outcome events or summary measures over time	8
Main results	16	(*a*) Give unadjusted estimates and, if applicable, confounder-adjusted estimates and their precision (eg, 95% confidence interval). Make clear which confounders were adjusted for and why they were included	8, 9
		(*b*) Report category boundaries when continuous variables were categorized	5, 6, 7
		(*c*) If relevant, consider translating estimates of relative risk into absolute risk for a meaningful time period	n/a
Other analyses	17	Report other analyses done—eg analyses of subgroups and interactions, and sensitivity analyses	9
**Discussion**			
Key results	18	Summarise key results with reference to study objectives	9, 10
**Limitations**			
Interpretation	20	Give a cautious overall interpretation of results considering objectives, limitations, multiplicity of analyses, results from similar studies, and other relevant evidence	9, 10, 11
Generalisability	21	Discuss the generalisability (external validity) of the study results	10, 11
**Other information**			
Funding	22	Give the source of funding and the role of the funders for the present study and, if applicable, for the original study on which the present article is based	12

*Give information separately for cases and controls in case-control studies and, if applicable, for exposed and unexposed groups in cohort and cross-sectional studies.

Note: An Explanation and Elaboration article discusses each checklist item and gives methodological background and published examples of transparent reporting. The STROBE checklist is best used in conjunction with this article (freely available on the Web sites of PLoS Medicine at http://www.plosmedicine.org/, Annals of Internal Medicine at http://www.annals.org/, and Epidemiology at http://www.epidem.com/). Information on the STROBE Initiative is available at www.strobe-statement.org.

For peer review only

BMJ Open

Do rapid BMI growth in childhood and early-onset obesity offer cardiometabolic protection to obese adults in mid-life? Analysis of a prospective cohort study of Danish men

Journal:	*BMJ Open*
Manuscript ID:	bmjopen-2014-004827.R1
Article Type:	Research
Date Submitted by the Author:	22-Mar-2014
Complete List of Authors:	Howe, Laura; University of Bristol, Social Medicine<br>Zimmermann, Esther; Institute of Preventive Medicine,<br>Weiss, Ram; The Hebrew University of Jerusalem, Braun School of Public Health<br>S�rensen, Thorkild; Institute of Preventive Medicine
**Primary Subject Heading**:	Epidemiology
Secondary Subject Heading:	Nutrition and metabolism, Cardiovascular medicine
Keywords:	Hypertension < CARDIOLOGY, Lipid disorders < DIABETES & ENDOCRINOLOGY, EPIDEMIOLOGY, NUTRITION & DIETETICS, PRIMARY CARE, PUBLIC HEALTH

SCHOLARONE™
Manuscripts

For peer review only

Do rapid BMI growth in childhood and early-onset obesity offer cardiometabolic protection to obese adults in mid-life? Analysis of a longitudinal cohort study of Danish men

Laura D Howe*[1], Esther Zimmermann[2], Ram Weiss[3], Thorkild IA Sørensen[2,4]

1 – MRC Integrative Epidemiology Unit at the University of Bristol, School of Social and Community Medicine, University of Bristol, Bristol, UK
2 – Institute of Preventive Medicine, Frederiksberg and Bispebjerg Hospitals, The Capital Region, Copenhagen, Denmark
3 – Braun School of Public Health, The Hebrew University of Jerusalem, Israel
4 - Novo Nordisk Center for Basic Metabolic Research, Faculty of Health and Medical Sciences, University of Copenhagen, Denmark

Key words: cardiovascular diseases, growth, metabolic syndrome, obesity, obese but metabolically healthy, metabolically abnormal but normal weight

*Corresponding author: Oakfield House, Oakfield Grove, Bristol, BS8 2BN, UK. Phone: +44 (0)117 3310134. Email: laura.howe@bristol.ac.uk

Article word length: 3,439

For peer review only

Abstract

Objectives: Some obese individuals have no cardiometabolic abnormalities; they are 'metabolically healthy, but obese' (MHO). Similarly, some non-obese individuals have cardiometabolic abnormalities, i.e. 'metabolically at risk, normal weight' (MANW). Previous studies have suggested early-onset obesity may be associated with MHO. We aimed to assess whether BMI in childhood and early-onset obesity are associated with MHO.

Setting: General population longitudinal cohort study, Denmark

Participants: From 362,200 young men (mean age 20) examined for Danish National Service between 1943-1977, all obese men (BMI≥31 kg/m^2, N=1,930) were identified along with a random 1% sample of the others (N=3,601). Our analysis includes 2,392 of these men attending a research clinic in mid-life (mean age 42). For 613 of these men, data on childhood BMI is available. We summarised childhood BMI growth (7-13 years) using a multilevel model. Early-onset obesity was defined as obesity at examination for National Service.

Outcome measurement: We defined metabolic health at the mid-life clinic as non-fasting serum cholesterol <6.6mmol/l, non-fasting glucose <8.39mmol/l and pulse pressure <48mmHg. Participants were categorised into four groups according to their obesity (BMI≥30 kg/m^2) and metabolic health in mid-life.

Results:

297 of 1,097 (27.1%) of obese men were metabolically healthy; 826 of 1,295 (63.8%) non-obese men had at least one metabolic abnormality. There was no evidence that rapid BMI growth in childhood or early-onset obesity was associated with either MHO or the MANW phenotype, e.g. amongst obese men in mid-life, the odds ratio for MHO comparing early-onset obesity with non-early-onset obesity was 0.97 (95% confidence interval 0.85 to 1.10).

Conclusions: We found no robust evidence that early onset obesity or rapid BMI growth in childhood is protective for cardiometabolic health.

Key words: metabolically healthy but obese, metabolic risk, cardiovascular health, obesity, child growth

For peer review only - http://bmjopen.bmj.com/site/about/guidelines.xhtml

Article summary: strengths and limitations of this study

1. This is the largest study to date to examine the question of whether childhood BMI and early onset obesity offer cardiometabolic protection to people who are obese as adults.
2. Using non-fasting measures of cholesterol and glucose and pulse pressure, we categorised men in mid life as either metabolically healthy, but obese (MHO), metabolically abnormal, obese (MAO), metabolically healthy, normal weight (MHNW) or metabolically abnormal, normal weight (MANW), and examined the relationships of childhood BMI growth and early onset obesity with this phenotype.
3. A limitation of this study is that our measures of cholesterol and glucose were from non-fasting blood samples. There is therefore potential misclassification in our measure of 'metabolically healthy'. We have tried to address this difficulty by using thresholds defined by percentiles of our data, selecting a relatively high cut-off for glucose in order to have reasonable specificity and sensitivity. Given that our estimated prevalence of MHO is within the range seen in previous studies, we feel that this is unlikely to have strongly biased our results.
4. We found no consistent evidence that rapid BMI growth in childhood, or early onset obesity, is associated with a favourable cardiometabolic profile, contradicting previous smaller studies that have examined this question.
5. Our findings imply that the notion of greater severity and metabolic impact of weight gain during adulthood is doubtful and that prevention measures should be implemented in the early childhood years.

For peer review only - http://bmjopen.bmj.com/site/about/guidelines.xhtml

Background:

Recent studies, including reviews[1-3], have drawn attention to the phenotype 'metabolically healthy, but obese' (MHO).[4;5] MHO individuals are defined as those who are obese but have a 'healthy' cardiometabolic profile; precise definitions have varied between studies, but tend to include one or more of insulin resistance, lipids, blood pressure, and markers of inflammation. The prevalence of MHO is estimated to be between 10-40% of obese people (with the remainder classified as metabolically at risk, obese, MAO).[1;6-9] Likewise, non-obese populations can be classified as either 'metabolically healthy, normal weight' (MHNW) or 'metabolically at risk, normal weight' (MANW).

Some studies have shown that MHO is associated with a reduced risk of mortality, cardiovascular disease events, type II diabetes, or a lower carotid intima-media thickness compared with MAO.[10-14] However, other studies, including a recent systematic review[15], have demonstrated similar or greater rates of mortality[15], or development of hypertension[16-18] and type II diabetes[17;18] in MHO compared with MAO or MHNW individuals, or high rates of conversion of MHO individuals to an MAO state[19;20], suggesting that MHO is an intermediate step in the development of MAO rather than a static condition, and that no level of obesity is healthy.[15;21;22]

Several factors have been shown to be positively associated with MHO, e.g. female gender, higher physical activity (though not in all studies[23]), greater fitness[24], longer sleep duration[23], and a favourable psychosocial profile.[1] There is also some evidence that childhood growth is associated with MHO; one small study (N=117 obese children, mean age 10.4 years) found higher birth weight and faster weight gain in infancy was associated with greater levels of circulating insulin and insulin resistance, whereas weight gain after age 4 years was negatively associated with insulin resistance.[25] Analyses of 499 obese adults in Helsinki, Finland, found that those who had the metabolic syndrome were similar in birth size compared with obese people who did not have metabolic syndrome, but were lighter and thinner between 2 and 11 years of age.[26] A further study of 43 postmenopausal women observed that an earlier age at onset of obesity is more common in MHO, with 48% of MHO women reporting an age of obesity onset <20 years compared with 29% of MAO women.[27] These findings are intriguing, given the well-established association between elevated BMI in childhood and greater risk of coronary heart disease in adulthood.[28;29] Research examining the influence of growth in childhood or early-onset obesity and MHO is, however, rare, since few studies have detailed data on both childhood growth and cardiometabolic health in adulthood.

For peer review only - http://bmjopen.bmj.com/site/about/guidelines.xhtml

In this paper, we take a cohort of men from Denmark, for whom data are available on childhood growth, early onset obesity, and cardiometabolic risk factors measured in mid-life. We examine the associations of early-onset obesity and trajectories of BMI growth in childhood (from seven to 13 years) with MHO, MAO, MHNW and MANW.

Materials and Methods:

Participants, exposure measurements and sample definition:

National service is compulsory in Denmark, and before entry into service, draftees attend a physical examination where their fitness to serve is assessed. In the 1970s, a study was initiated to make use of the anthropometric data collected at these examinations. From the 362,200 men examined in the metropolitan area of Copenhagen between 1943-1977, all 1,930 men who were obese (defined as 35% overweight relative to a Scandinavian standard in use at the time, equivalent to a body mass index (BMI) ≥31kg/m^2) and a random sample of 1% of the others (N=3,601) were selected to form a cohort study. Only 3% of men eligible for national service were not examined for medical reasons, with obesity not being a condition that allowed exemption.[30] A further 2% of men were not examined because they had volunteered for service before the age of 18 years. Height was measured without shoes, and weight in only underwear. The mean age at examination was 20 years, range 18-31.

Two re-examinations were undertaken of this cohort, firstly in 1982-1984 (between 4-40 years after the draft board examination, mean age 35 years, range 22-64) and secondly in 1991-1994 (mean age of participants 45.6 years, range 33-75). All of the cohort members who were obese at draft board examination and (for logistical reasons) half of the controls were invited to these clinics, which were attended by 2,075 and 1,703 participants respectively. Previous analyses of this cohort have shown that lower BMI and higher intelligence, educational level, social class and age were associated with a greater probability of attending follow-up clinics.[31] Further details of this cohort are published elsewhere.[30;32-35] As part of a separate study[36;37], anthropometric data extracted from school health records between ages 7 and 13 are available for a subset of individuals. Participants gave informed consent, and the protocol was consistent with the principles of the Declaration of Helsinki.

For our main analysis (association between early onset obesity and obesity-metabolic status in mid-life), we included all participants who attended either the first or second follow-up clinic and had their BMI, systolic and diastolic blood pressure (SBP/DBP), cholesterol and glucose measured. We

For peer review only - http://bmjopen.bmj.com/site/about/guidelines.xhtml

used data from the second follow-up clinic where available, or the first follow-up clinic for participants attending the first but not second assessment. For our secondary analysis of the association between childhood growth trajectories and obesity-metabolic status in mid-life, this sample was further restricted to participants with one or more BMI measure from school health records between 7-13 years.

Exposure measurements:

Obesity status (BMI ≥31kg/m^2) in early adulthood (at entry into National Service, mean age 20 years) determines our measurement of early-onset obesity.

We used a multilevel model to estimate BMI at age 7 and the linear change in BMI between 7-13 years for all participants with one or more BMI measure. Multilevel models are an appropriate tool for the analysis of longitudinal data, since they allow for the non-independence of repeated measures on the same individual, and can estimate a full trajectory for all participants with one or more measurement under a missing at random assumption.[38;39] The multilevel model was run in MLwiN version 2.26[40] via Stata version 12[41], using the command runmlwin.[42] Full details of the model are provided in online supplementary material. From the multilevel model, we derived predicted BMI at age 7 and predicted linear change in BMI between 7-13 years; these variables were used as exposures in our analyses.

Outcome measurements:

At each follow-up clinic, height was measured without shoes and weight was measured in light clothes and without shoes. SBP and DBP were measured three times with the participant at rest; the mean of the 3 measures was used. Total serum glucose and cholesterol were assessed from non-fasting blood samples (examinations were undertaken during day time, and the participants were not requested to skip breakfast or lunch) drawn from a vein after light stasis. Samples were placed in a box containing ice water, and within two hours centrifuged at 3,000rpm for ten minutes. The plasma was stored at 4°C and analysed the following day. Cholesterol was measured enzymatically using the CHOD-PAP method at the first examination and high-performance CHOD-PAP at the second (Boehringer Mannheim). Glucose was assayed using the hexokinase/G6P-DH assy. Blood samples remained at room temperature for 20-60 minutes, after which the whole blood from the EDTA tube was placed in a plastic tube and frozen at -20°C. To control for treatment effects, a constant of 10mmHg was added to SBP and 5mmHg to DBP for individuals reporting treatment with antihypertensive medication (n=156); these values are consistent with the effects of antihypertensive medications, and this method has been shown to be a reasonable approach for

For peer review only - http://bmjopen.bmj.com/site/about/guidelines.xhtml

reducing treatment bias and has been used in previous studies.[43-45] Pulse pressure, identified in previous research as an important independent risk factor for cardiovascular disease[46], was defined as SBP-DBP.

For each cardiometabolic risk factor (SBP, DBP, pulse pressure, glucose and cholesterol), a measure of the level of the risk factor given what would be predicted from age and BMI was created by regressing the risk factor on age and BMI at the time of assessment, and standardising the resulting residuals to have a mean of zero and variance of one. Thus for these measures a value of zero would mean that the level of the risk factor is what would be predicted given age and BMI, positive values would mean higher than expected levels, and vice versa for negative values.

Various definitions of MHO have been used in previous studies.[1;2] Since the blood samples in our study were non-fasting, standard cut-offs based on fasting samples would be too low. We therefore opted to define cut-offs for cholesterol and glucose based on percentiles of the study-population distribution. For cholesterol, we deemed levels above the 75th centile (6.6mmol/l) to be high. Fluctuations in glucose are greater than those in cholesterol, so a higher threshold is necessary in order to obtain a measure that is reasonably sensitive but also has sufficient specificity. Therefore we defined high glucose levels as above the 95% centile of non-obese participants (8.39mmol/l). In line with previous research[46], we defined high pulse pressure as above 48. 'Metabolically healthy' was defined as having cholesterol, glucose and pulse pressure levels below these thresholds; participants with one or more measurement above these thresholds were deemed 'metabolically at risk'. Individuals were classified according to their obesity status in mid-life (obesity defined as ≥30 kg/m^2) and metabolic status, such that individuals were MHO, MAO, MHNW or MANW. As sensitivity analyses, we repeated our analyses using lower cut-points for glucose (≤5.6mmol/l) and cholesterol (<5.2mmol/l), and using systolic and diastolic blood pressure instead of pulse pressure.

Other data:

The occupation of participants' fathers was self-reported in questionnaires and coded as manual or non-manual. A binary indicator of participants' smoking behaviour (any versus no smoking) was created from self-reported data.

Statistical analysis:

The associations of early onset obesity and childhood growth with the cardiometabolic risk factors and standardised residuals from regression of cardiometabolic risk factors on age and BMI were

For peer review only - http://bmjopen.bmj.com/site/about/guidelines.xhtml

assessed using linear regression. The associations of early onset obesity and childhood growth with MHO (compared with MAO) or with MHNW (compared with MANW) were assessed using logistic regression. As a sensitivity analysis, we assessed whether our findings are robust to adjustment for father's social class and participants' smoking status.

Results:

2,392 participants were included in our analyses; of these, data from the second follow-up assessment were available for 1,691 and from the first assessment for a further 701 (**Table 1**).

Prevalence of obesity and metabolic phenotypes:

Amongst the 2,392 participants, 1,295 were not obese. Of these, 469 (36.2%) were classified as MHNW, and 826 (63.8%) as MANW (**Table 1**). Thus 63.8% of normal weight individuals had one or more metabolic abnormality. Of the 1,097 participants who were obese at follow-up, 297 (27.1%) were MHO.

Compared with MANW individuals, MHNW individuals tended to be younger (mean and standard deviation (SD) for age 44.93 (10.89) and 42.09 (8.40) years respectively) and thinner (mean and SD for BMI 25.55 (2.69) and 24.82 (2.85) years) (**Table 2**). MAO had higher BMI compared with MHO individuals (mean and SD BMI 36.49 (5.10) and 35.79 (4.53) kg/m^2), but there was little difference in age (**Table 2**).

Childhood growth trajectories:

Differences in BMI between participants who were obese and non-obese at draft board examination were already evident by age 7. The mean BMI at age 7 and rate of BMI change between 7-13 years were higher in those who were obese at draft board (**Web Table 1**). The multilevel model had good fit to the data (**Web Table 2** and **Web Figures 1-3**).

Associations of childhood growth and early onset obesity with cardiometabolic health and obesity/metabolic phenotypes in mid-life:

Those with early onset obesity (i.e. those who were obese at draft board examination) had a BMI in mid-life on average 9.65 kg/m^2 (95% CI 9.27 to 10.03) greater than those who were not obese at the draft board examination. Greater BMI at age 7, faster rate of BMI increase between 7-13 years and

For peer review only - http://bmjopen.bmj.com/site/about/guidelines.xhtml

early onset obesity were all associated with greater SBP, DBP and pulse pressure, and greater glucose levels in mid-life (**Table 3**). These associations tended to increase in magnitude after adjustment for age at the research clinic and year of draft board examination. In our analyses, the associations of childhood growth and early onset obesity with levels of cholesterol were weaker than for the other cardiometabolic risk factors.

There was very little evidence of associations of childhood growth and early onset obesity with standardised scores of the cardiometabolic risk factors that account for age and BMI at the time of assessment (**Table 4**). There was no evidence of associations for the standardised scores of SBP or DBP. For standardised scores of glucose, early onset obesity and faster rate of BMI increase between 7-13 years were both associated with higher scores (no association was seen for BMI at 7 years). For example, after adjustment for year at draft board, early onset obesity was associated with a 0.16 SD higher standardised score of glucose (95% CI 0.08 to 0.24), and a 1 kg/m^2 per year increase in the rate of BMI growth between 7-13 years (equivalent to approximately 2 SD of the rate of growth) was associated with a 0.26 SD higher standardised score (95% CI 0.03 to 0.49). Conversely, early onset obesity, greater BMI at age 7 and faster rate of BMI increase between 7-13 years were all associated with lower standardised scores of cholesterol. For example, after adjustment for year of draft board examination, early onset obesity was associated with a -0.19 lower standardised score for cholesterol (95% CI -0.27 to -0.11) and a 1 kg/m^2 per year increase in the rate of BMI growth between 7-13 years was associated with a -0.12 lower standardised score (95% CI -0.35 to 0.10). Thus early onset obesity and faster rates of BMI increase in childhood are associated with a higher glucose level but lower cholesterol level than would be predicted from age and adult BMI.

Among those who were not obese at the follow-up clinic, early onset obesity was associated with slightly lower odds of being MHNW compared with MANW, but the confidence interval was wide and included the null value (odds ratio 0.76, 95% CI 0.54 to 1.07) (**Table 5**). There was no consistent relationship between childhood BMI trajectories and MHNW compared with MANW.

Among those who were obese in mid-life, there was a weak association between early onset obesity and lower odds of being MHO compared with MAO; odds ratio 0.69, 95% CI 0.46 to 1.04. There was no association between BMI at age 7 or BMI increases between 7-13 years and MHO (**Table 5**). Results were unchanged when adjusted for father's social class and participant's smoking (**Web Table 3**).

For peer review only - http://bmjopen.bmj.com/site/about/guidelines.xhtml

Associations of early onset obesity and child growth with MHO remained similar if lower thresholds for defining abnormal cholesterol and glucose were used, or if systolic and diastolic blood pressure were used in the definition of metabolically healthy instead of pulse pressure (results available from authors on request).

Discussion:

We found little consistent evidence to support the hypothesis that rapid BMI growth between 7-13 years and early onset obesity are associated with a protective cardiometabolic effect. Early onset obesity and faster rates of BMI increase in childhood were associated with a higher glucose level but lower cholesterol level than would be predicted from age and adult BMI. No associations were seen for SBP or DBP levels or pulse pressure adjusted for concurrent age and BMI. There was also no consistent evidence that early onset obesity or rates of BMI change in childhood were associated with being 'metabolically healthy' – early onset obesity was weakly associated with lower odds of being MHO, but no associations were seem for BMI at age 7 or rate of BMI change between 7-13 years. Our findings contrast with a study of 43 postmenopausal women, which observed that 48% of MHO women reported an age of obesity onset <20 years compared with 29% of MAO women.[27] The earliest available childhood BMI measures in our cohort are from age 7; thus it is possible that BMI changes at earlier stages could be associated with being metabolically healthy, and we are thus unable to make direct comparisons between our results and those of the studies looking at birth size and weight gain in early childhood in relation to MHO.[25;26] Our findings are supported by some previous research on this cohort, which has found that the men who were obese at their draft board examination in early adulthood experience a doubling of all-cause mortality risk[47], as well as increased risks of mortality from cardiovascular diseases and a wide range of other specific causes.[48] However, other analyses of this cohort have demonstrated that obese men, maintaining their weight since the draft board examination in early adulthood, had lower risk of impaired glucose tolerance than non-obese men in early adulthood who became similarly obese by age 51.[49]

In this cohort of Danish men, 27.1% of participants who were obese at follow-up were classified as MHO defined by having non-fasting cholesterol <6.6mmol/l, non-fasting glucose <8.39mmol/l and pulse pressure <48mmHg. This is within the range of 10-40% prevalence reported in previous studies.[1;6-9] A large study of over 5000 adults in the National Health and Nutrition Examination Surveys from the USA found a prevalence of MHO of 51.3%.[50] However, this study differed from ours

For peer review only - http://bmjopen.bmj.com/site/about/guidelines.xhtml

in two important ways; firstly by considering overweight participants rather than obese, and secondly by considering metabolically at risk to be two or more abnormalities, whereas we assumed one or more abnormality conferred risk.

Notably, a very high proportion of non-obese participants were classified as MANW: 63.8%. Analysis of data from a Chinese cohort also found a high prevalence of cardiometabolic abnormalities in non-obese adults[51], leading the authors to conclude that focusing cardiovascular screening only on obese individuals would miss a large proportion of the at risk population. The NHANES data also found that 23.5% of non-overweight participants had at least two metabolic abnormalities.[50] In our study, the mean BMI in the non-obese group was high – 24.82 kg/m^2 in the MHNW group and 25.55 in the MANW group. Thus although not obese, many of these participants were overweight.

Our analysis included 2,392 individuals, 1,097 of whom were obese, making it larger than most existing studies on MHO.[1;2] We were only able to include a subset of these individuals (N=613) in our analysis of the association between childhood BMI growth and later metabolic health. This is a larger sample size than the former studies on this topic[25;27], but we may still have had limited power to detect an association. We also were unable to examine the influence of growth before age 7 or of puberty. Furthermore, some of our participants were quite young at the follow-up examinations, and so associations may emerge in later life as cardiovascular risk develops. Our measure of early-onset obesity is based on obesity at a mean age of 20 years; whilst we feel that this is an appropriate definition of early-onset obesity in this population, who grew up at a time when childhood obesity was very rare, studies in younger cohorts may wish to consider earlier ages to define 'early' obesity. Our measures of adiposity are based on BMI, which is an imperfect measure of fatness, particularly in children. However, even in childhood, BMI has been shown to have similar associations with cardiovascular risk factors as directly-determined fat mass.[52]

We did not have data on insulin or on lipids other than total cholesterol, or other measures that may be stronger predictors of cardiovascular events, and therefore we could not incorporate these into our definitions of MHO, MAO, MHNW and MANW. Our measures of cholesterol and glucose were from non-fasting blood samples. There is therefore potential misclassification in our measure of 'metabolically healthy'. We have tried to address this difficulty by using thresholds defined by percentiles of our data. Glucose levels are likely to fluctuate more in non-fasting samples than cholesterol levels[53], and we therefore selected a relatively high cut-off for glucose in order to have reasonable specificity and sensitivity. Given that our estimated prevalence of MHO is within the

For peer review only - http://bmjopen.bmj.com/site/about/guidelines.xhtml

range seen in previous studies, we feel that this is unlikely to have strongly biased our results, which remained similar if lower cut-points were used for cholesterol and glucose.

We found no consistent evidence that rapid BMI growth in childhood, or early onset obesity, is associated with a favourable cardiometabolic profile, contradicting previous smaller studies that have examined this question. Our findings imply that the notion of greater severity and metabolic impact of weight gain during adulthood is doubtful and that prevention measures should be implemented in the early childhood years.

For peer review only

For peer review only - http://bmjopen.bmj.com/site/about/guidelines.xhtml

Contributions of each author: The study was conceived by LDH and TIAS; the study was designed by LDH with input from TIAS and RW; LDH carried out statistical analyses and wrote the first draft of the manuscript; all authors contributed to critical revisions of the manuscript and approved the final submitted version

Data sharing statement: statistical code can be requested from the corresponding author. For details of data access and collaboration policy, please contact Thokild Sørensen: tias@sund.ku.dk

Funding statement: This work was supported by a UK Medical Research Fellowship to LDH (G1002375); the funders had no role in the design or conduct of the study or the decision to publish.

Competing interests statement: Competing interests: None declared

For peer review only

Table 1: Sample characteristics, mean (SD) or % characteristics of participants included in analyses

	**Participants included in analysis of the association between early-onset obesity and metabolic health in mid-life**			**Participants included in analysis of the association between childhood growth and metabolic health in mid-life**		
	Participants attending second follow-up in adulthood	Participants attending first follow-up in adulthood but not the second follow-up	Combined whole sample	Participants attending second follow-up in adulthood	Participants attending first follow-up in adulthood but not the second follow-up	Combined whole sample
	**N=1,691**	**N=701**	**N=2,392**	**N=420**	**N=193**	**N=613**
Age (years)	45.61 (7.93)	34.81 (8.38)	42.44 (9.45)	48.01 (6.82)	36.34 (6.90)	44.34 (8.73)
Number (%) obese in early adulthood*	784 (46.4%)	346 (49.4%)	1,130 (47.2%)	189 (45.0%)	95 (49.2%)	284 (46.3%)
Body mass index (kg/m^2)	30.57 (6.69)	29.76 (6.87)	30.34 (6.75)	30.52 (6.79)	31.00 (7.74)	30.67 (7.10)
Systolic blood pressure (mmHg)	142.37 (18.12)	138.50 (16.18)	141.89 (18.32)	143.64 (18.75)	138.08 (16.33)	141.89 (18.19)
Diastolic blood pressure (mmHg)	92.81 (11.41)	86.45 (12.25)	91.27 (12.31)	92.29 (10.81)	89.27 (11.70)	91.34 (11.18)
Pulse pressure (mmHg)	49.96 (13.26)	52.22 (12.38)	50.62 (13.04)	51.36 (13.73)	48.80 (11.91)	50.55 (13.23)
Glucose (mmol/l)	6.48 (2.95)	6.57 (2.42)	6.51 (2.80)	6.56 (3.14)	6.65 (2.52)	6.59 (2.96)
Cholesterol (mmol/l)	6.10 (1.28)	5.25 (1.22)	5.85 (1.32)	6.11 (1.20)	5.34 (1.24)	6.86 (1.26)
*Number (%) with metabolic phenotypes ***						
NON-OBESE AT MID-LIFE FOLLOW-UP	*N=903*	*N=392*	*N=1,295*	*N=235*	*N=102*	*N=337*
Metabolically healthy	328 (36.3%)	141 (36.0%)	469 (36.2%)	81 (34.5%)	49 (48.0%)	130 (38.6%)
Metabolically at risk	575 (63.7%)	251 (64.0%)	826 (63.8%)	154 (65.5%)	53 (52.0%)	207 (61.4%)
OBESE AT MID-LIFE FOLLOW-UP	*N=788*	*N=309*	*N=1,097*	*N=185*	*N=91*	*N=276*
Metabolically healthy, but obese	221 (28.0%)	76 (24.6%)	297 (27.1%)	48 (25.9%)	28 (30.8%)	76 (27.5%)
Metabolically at risk, obese	567 (72.0%)	233 (75.4%)	800 (72.9%)	137 (74.1%)	63 (69.2%)	200 (72.5%)

*Defined as 35% overweight relative to a Scandinavian standard in use at the time, equivalent to a body mass index (BMI) $\geq 31 kg/m^2$

**Metabolically healthy is defined by having pulse pressure (systolic – diastolic blood pressure) <48mmHg, glucose <8.39mmol/l, and cholesterol <6.6mmol/l; i.e. any one or more metabolic abnormality confers 'metabolically at-risk' status. Obesity at the mid-life follow-up is defined by $BMI \geq 30 kg/m^2$

For peer review only - http://bmjopen.bmj.com/site/about/guidelines.xhtml

Table 2: Characteristics of participants within each metabolic and obesity category

	Metabolically healthy, normal weight (MHNW)*	Metabolically at risk, normal weight (MANW)*	Metabolically healthy, but obese (MHO)*	Metabolically at risk, obese (MAO)*
Mean (SD)				
	N=469	N=826	N=297	N=800
Age (years)	42.09 (8.40)	44.93 (10.89)	40.15 (7.33)	40.93 (8.53)
BMI (kg/m^2)	24.82 (2.85)	25.55 (2.69)	35.79 (4.53)	36.49 (5.10)
Systolic blood pressure (mmHg)	126.60 (10.37)	142.97 (16.98)	134.70 (10.74)	152.41 (18.22)
Diastolic blood pressure (mmHg)	86.85 (9.21)	88.00 (12.34)	95.21 (9.77)	95.77 (12.76)
Pulse pressure (mmHg)	39.74 (5.23)	54.97 (11.97)	39.49 (5.67)	56.64 (12.73)
Cholesterol (mmol/l)	5.26 (0.87)	6.06 (1.40)	5.39 (0.82)	6.15 (1.45)
Glucose (mmol/l)	5.62 (0.87)	6.29 (2.30)	5.76 (0.74)	7.53 (3.95)
Number (%) that were obese in early adulthood**	61 (13.0%)	118 (14.3%)	253 (85.2%)	698 (87.3%)

* Metabolically healthy is defined by having pulse pressure (systolic – diastolic blood pressure) <48mmHg, glucose <8.39mmol/l, and cholesterol <6.6mmol/l; i.e. any one or more metabolic abnormality confers 'metabolically at-risk' status. Obesity in mid-life is defined by BMI≥30kg/m^2

**Obesity in early adulthood is defined as 35% overweight relative to a Scandinavian standard in use at the time, equivalent to a body mass index (BMI) ≥31kg/m^2

For peer review only

For peer review only - http://bmjopen.bmj.com/site/about/guidelines.xhtml

Table 3: Linear regression coefficients (with 95% confidence intervals) for the association of early onset obesity and childhood BMI trajectories with cardiovascular risk factors measured in mid-life.

Model*	Exposure	Outcome					
		BMI (kg/m^2)	Systolic blood pressure (mmHg)	Diastolic blood pressure (mmHg)	Pulse pressure (mmHg)	Glucose (mmol/l)	Cholesterol (mmol/l)
Full sample, N=2,329							
1	Early onset obesity**	9.65 (9.27 to 10.03) p<0.001	7.70 (6.27 to 9.14) p<0.001	5.31 (4.35 to 6.28) p<0.001	2.39 (1.35 to 3.44) p<0.001	1.14 (0.92 to 1.36) p<0.001	-0.14 (-0.25 to -0.04) p=0.01
2		9.76 (9.36 to 10.16) p<0.001	10.28 (8.82 to 11.74) p<0.001	6.02 (5.01 to 7.02) p<0.001	4.27 (3.21 to 5.33) p<0.001	1.30 (1.07 to 1.53) p<0.001	-0.07 (-0.18 to 0.04) p=0.19
Subsample with childhood growth data, N=613							
1	BMI at 7 years (kg/m^2)	1.81 (1.60 to 2.02) p<0.001	1.37 (0.72 to 2.02) p<0.001	0.72 (0.32 to 1.12) p<0.001	0.65 (0.17 to 1.13) p=0.01	0.12 (0.02 to 0.23) p=0.03	-0.04 (-0.09 to 0.00) p=0.08
2		1.75 (1.53 to 1.97) p<0.001	1.82 (1.17 to 2.47) p<0.001	0.85 (0.44 to 1.26) p<0.001	0.97 (0.49 to 1.45) p<0.001	0.13 (0.02 to 0.24) p=0.02	-0.02 (-0.06 to 0.03) p=0.50
1	BMI change 7-13 years (kg/m^2 per year)	10.95 (10.01 to 11.89) p<0.001	8.47 (5.26 to 11.67) p<0.001	4.53 (2.55 to 6.51) p<0.001	3.93 (1.57 to 6.30) p=0.001	1.31 (0.78 to 1.83) p<0.001	-0.20 (-0.42 to 0.03) p=0.09
2		8.75 (7.59 to 9.91) p<0.001	9.69 (5.76 to 13.63) p<0.001	4.76 (2.27 to 7.25) p<0.001	4.93 (2.00 to 7.87) p=0.001	1.58 (0.91 to 2.24) p<0.001	0.05 (-0.22 to 0.33) p=0.71

*Model details: Model 1 is unadjusted; Model 2 is adjusted for age at outcome assessment and year of draft board examination, and the coefficient for change in BMI between 7-11 years is additionally adjusted for BMI at 7 years as predicted by the multilevel model

**Defined as 35% overweight relative to a Scandinavian standard in use at the time, equivalent to a body mass index (BMI) ≥31kg/m^2 in early adulthood, mean age 20 years

For peer review only - http://bmjopen.bmj.com/site/about/guidelines.xhtml

Table 4: Linear regression coefficients (with 95% confidence intervals) for the association of early onset obesity and childhood BMI trajectories with levels of cardiovascular risk factors in mid-life adjusted for age and BMI in mid-life.

Model*	Exposure	Outcomes** (standard deviations, given age and concurrent BMI)				
		Systolic blood pressure	Diastolic blood pressure	Pulse pressure	Glucose	Cholesterol
Full sample, N=2,329						
1	Early onset obesity***	0.02 (-0.06 to 0.10) p=0.62	-0.01 (-0.09 to 0.07) p=0.78	0.04 (-0.04 to 0.12) p=0.38	0.13 (0.05 to 0.21) p=0.002	-0.13 (-0.21 to -0.05) p=0.002
2		0.05 (-0.04 to 0.13) p=0.28	-0.06 (-0.14 to 0.03) p=0.18	0.11 (0.03 to 0.19) p=0.01	0.16 (0.08 to 0.24) p<0.001	-0.19 (-0.27 to -0.11) p<0.001
Subsample with childhood growth data, N=613						
1	BMI at 7 years (kg/m^2)	0.00 (-0.04 to 0.04) p=0.97	-0.01 (-0.05 to 0.03) p=0.57	0.01 (-0.03 to 0.05) p=0.61	-0.01 (-0.05 to 0.02) p=0.42	-0.03 (-0.07 to 0.00) p=0.07
2		0.00 (-0.03 to 0.04) p=0.89	-0.01 (-0.05 to 0.02) p=0.47	0.01 (-0.02 to 0.05) p=0.49	-0.01 (-0.05 to 0.02) p=0.50	-0.04 (-0.08 to -0.01) p=0.02
1	BMI change 7-13 years (kg/m^2 per year)	0.03 (-0.15 to 0.21) p=0.75	-0.04 (-0.22 to 0.14) p=0.69	0.07 (-0.11 to 0.25) p=0.46	0.11 (-0.07 to 0.29) p=0.24	-0.15 (-0.33 to 0.03) p=0.10
2		0.05 (-0.18 to 0.28) p=0.65	-0.02 (-0.25 to 0.20) p=0.83	0.07 (-0.16 to 0.31) p=0.52	0.26 (0.03 to 0.49) p=0.03	-0.12 (-0.35 to 0.10) p=0.28

The outcome in these analyses is a measure of the level of the cardiovascular risk factor (SBP, DBP, pulse pressure, glucose and cholesterol) given what would be predicted from age and BMI; this was created by regressing the risk factor on age and BMI at the time of assessment, and standardising the resulting residuals to have a mean of zero and variance of one. Thus for these measures a value of zero would mean that the level of the risk factor is what would be predicted given age and BMI, positive values would mean higher than expected levels, and vice versa for negative values.

*Model details: Model 1 is unadjusted; Model 2 is adjusted for year of draft board examination, in model 2 the coefficient for change in BMI between 7-11 years is additionally adjusted for BMI at 7 years as predicted by the multilevel model

**Cardiovascular risk factors were regressed on concurrent BMI and age, and standardised residuals (mean of zero, variance of one) from those regressions are used as the outcomes in these analyses. Thus a positive value of these outcomes means that an individual has a higher level of that outcome than would be expected given their age and BMI, and a negative value means the opposite, lower level of the outcome given their age and BMI

***Defined as 35% overweight relative to a Scandinavian standard in use at the time, equivalent to a body mass index (BMI) ≥31kg/m^2 in early adulthood, mean age 20 years

For peer review only

Table 5: Odds ratios (with 95% confidence intervals) for the association of obese status in early adulthood and growth in early childhood with metabolic status in mid-life; stratified by mid-life obesity status

Model*	Metabolically healthy, normal weight**	Metabolically at risk, normal weight**	Metabolically healthy, but obese**	Metabolically at risk, obese**
Full sample, N=2,329				
	N=469	*N=826*	*N=297*	*N=800*
**Early onset obesity*****				
1	0.90 (0.64 to 1.25) p=0.52	1 (ref)	0.84 (0.57 to 1.23) p=0.37	1 (ref)
2	0.76 (0.54 to 1.07) p=0.12	1 (ref)	0.69 (0.46 to 1.04) p=0.08	1 (ref)
Subsample with childhood growth data, N=613				
	N=130	*N=207*	*N=76*	*N=200*
**BMI at 7 (kg/m^2)**				
1	0.97 (0.85 to 1.10) p=0.60	1 (ref)	0.99 (0.88 to 1.13) p=0.92	1 (ref)
2	0.93 (0.81 to 1.07) p=0.32	1 (ref)	0.97 (0.85 to 1.10) p=0.65	1 (ref)
**BMI change 7-13 (kg/m^2 per year)**				
1	0.85 (0.41 to 1.70) p=0.62	1 (ref)	1.50 (0.73 to 3.05) p=0.27	1 (ref)
2	0.66 (0.25 to 1.72) p=0.39	1 (ref)	1.40 (0.61 to 3.48) p=0.38	1 (ref)

Coefficients are odds ratios (95% confidence intervals) comparing obese in early adulthood with non-obese in early adulthood, or a one unit increase in BMI at age 7 or the linear rate of change in BMI between 7 and 13 years.

*Model details: Model 1 is unadjusted; Model 2 is adjusted for age at outcome assessment and year of draft board examination. In model 2 the coefficient for change in BMI between 7-13 years is additionally adjusted for BMI at 7 years as predicted by the multilevel model

** Metabolically healthy is defined by having pulse pressure (systolic – diastolic blood pressure) <48mmHg, glucose <8.39mmol/l, and cholesterol <6.6mmol/l; i.e. any one or more metabolic abnormality confers 'metabolically at-risk' status. Obesity in mid-life is defined by BMI≥30kg/m^2

***Defined as 35% overweight relative to a Scandinavian standard in use at the time, equivalent to a body mass index (BMI) ≥31kg/m^2 in early adulthood, mean age 20 years

For peer review only

Reference List

(1) Primeau V, Coderre L, Karelis AD, et al. Characterizing the profile of obese patients who are metabolically healthy. *Int J Obes* 2011; 35(7):971-981.

(2) Denis GV, Obin MS. 'Metabolically healthy obesity': Origins and implications. *Mol Aspects Med* 2012.

(3) Ahima RS, Lazar MA. The health risk of obesity - better metrics imperative. *Science* 2013; 341:856.

(4) Sorensen TI, Virtue S, Vidal-Puig A. Obesity as a clinical and public health problem: is there a need for a new definition based on lipotoxicity effects? *Biochim Biophys Acta* 2010; 1801(3):400-404.

(5) Sorensen TI. Obesity defined as excess storage of inert triglycerides--do we need a paradigm shift? *Obes Facts* 2011; 4(2):91-94.

(6) Karelis AD, Brochu M, Rabasa-Lhoret R. Can we identify metabolically healthy but obese individuals (MHO)? *Diabetes Metab* 2004; 30(6):569-572.

(7) Karelis AD, Brochu M, Rabasa-Lhoret R, et al. Clinical markers for the identification of metabolically healthy but obese individuals. *Diabetes Obes Metab* 2004; 6(6):456-457.

(8) Karelis AD, St-Pierre DH, Conus F, et al. Metabolic and body composition factors in subgroups of obesity: what do we know? *J Clin Endocrinol Metab* 2004; 89(6):2569-2575.

(9) Karelis AD. Metabolically healthy but obese individuals. *Lancet* 2008; 372(9646):1281-1283.

(10) Meigs JB, Wilson PW, Fox CS, et al. Body mass index, metabolic syndrome, and risk of type 2 diabetes or cardiovascular disease. *J Clin Endocrinol Metab* 2006; 91(8):2906-2912.

(11) Durward CM, Hartman TJ, Nickols-Richardson SM. All-Cause Mortality Risk of Metabolically Healthy Obese Individuals in NHANES III. *J Obes* 2012; 2012:460321.

(12) Hamer M, Stamatakis E. Metabolically healthy obesity and risk of all-cause and cardiovascular disease mortality. *J Clin Endocrinol Metab* 2012; 97(7):2482-2488.

(13) Calori G, Lattuada G, Piemonti L, et al. Prevalence, metabolic features, and prognosis of metabolically healthy obese Italian individuals: the Cremona Study. *Diabetes Care* 2011; 34(1):210-215.

(14) Li S, Chen W, Srinivasan SR, et al. Relation of childhood obesity/cardiometabolic phenotypes to adult cardiometabolic profile: the Bogalusa Heart Study. *Am J Epidemiol* 2012; 176 Suppl 7:S142-S149.

(15) Kramer CK, Zinman B, Retnakaran R. Are metabolically healthy overweight and obesity benign conditions?: A systematic review and meta-analysis. *Ann Intern Med* 2013; 159(11):758-769.

For peer review only

For peer review only - http://bmjopen.bmj.com/site/about/guidelines.xhtml

(16) Lee SK, Kim SH, Cho GY, et al. Obesity phenotype and incident hypertension: a prospective community-based cohort study. *J Hypertens* 2013; 31(1):145-151.

(17) Hwang LC, Bai CH, Sun CA, et al. Prevalence of metabolically healthy obesity and its impacts on incidences of hypertension, diabetes and the metabolic syndrome in Taiwan. *Asia Pac J Clin Nutr* 2012; 21(2):227-233.

(18) Chang Y, Ryu S, Suh BS, et al. Impact of BMI on the incidence of metabolic abnormalities in metabolically healthy men. *Int J Obes (Lond)* 2012; 36(9):1187-1194.

(19) Appleton SL, Seaborn CJ, Visvanathan R, et al. Diabetes and Cardiovascular Disease Outcomes in the Metabolically Healthy Obese Phenotype: A cohort study. *Diabetes Care* 2013.

(20) Bradshaw PT, Monda KL, Stevens J. Metabolic syndrome in healthy obese, overweight, and normal weight individuals: The atherosclerosis risk in communities study. *Obesity (Silver Spring)* 2013; 21(1):203-209.

(21) Hill JO, Wyatt HR. The myth of healthy obesity. *Ann Intern Med* 2013; 159(11):789-790.

(22) Thomsen M, Nordestgaard BG. Myocardial infarction and ischemic heart disease in overweight and obesity with and without metabolic syndrome. *JAMA Intern Med* 2014; 174(1):15-22.

(23) Hankinson AL, Daviglus ML, Van HL, et al. Diet composition and activity level of at risk and metabolically healthy obese American adults. *Obesity (Silver Spring)* 2012.

(24) Ortega FB, Lee DC, Katzmarzyk PT, et al. The intriguing metabolically healthy but obese phenotype: cardiovascular prognosis and role of fitness. *Eur Heart J* 2013; 34(5):389-397.

(25) Bouhours-Nouet N, Dufresne S, de Casson FB, et al. High birth weight and early postnatal weight gain protect obese children and adolescents from truncal adiposity and insulin resistance: metabolically healthy but obese subjects? *Diabetes Care* 2008; 31(5):1031-1036.

(26) Salonen MK, Kajantie E, Osmond C, et al. Role of childhood growth on the risk of metabolic syndrome in obese men and women. *Diabetes Metab* 2009; 35(2):94-100.

(27) Brochu M, Tchernof A, Dionne IJ, et al. What are the physical characteristics associated with a normal metabolic profile despite a high level of obesity in postmenopausal women? *J Clin Endocrinol Metab* 2001; 86(3):1020-1025.

(28) Baker JL, Olsen LW, Sorensen TI. Childhood body-mass index and the risk of coronary heart disease in adulthood. *New England Journal of Medicine* 2007; 357:2329-2337.

(29) Owen CG, Whincup PH, Orfei L, et al. Is body mass index before middle age related to coronary heart disease risk in later life? Evidence from observational studies. *International Journal of Obesity* 2009; 33:866-877.

(30) Sorensen TI, Sonne-Holm S. Mortality in extremely overweight young men. *J Chronic Dis* 1977; 30(6):359-367.

For peer review only - http://bmjopen.bmj.com/site/about/guidelines.xhtml

(31) Sonne-Holm S, Sorensen TI, Jensen G, et al. Influence of fatness, intelligence, education and sociodemographic factors on response rate in a health survey. *J Epidemiol Community Health* 1989; 43(4):369-374.

(32) Sonne-Holm S, Sorensen TI, Christensen U. Risk of early death in extremely overweight young men. *Br Med J (Clin Res Ed)* 1983; 287(6395):795-797.

(33) Sonne-Holm S, Sorensen TI, Jensen G, Schnohr P. Long-term changes of body weight in adult obese and non-obese men. *Int J Obes* 1990; 14(4):319-326.

(34) Sonne-Holm S, Sorensen TI, Jensen G, et al. Independent effects of weight change and attained body weight on prevalence of arterial hypertension in obese and non-obese men. *BMJ* 1989; 299(6702):767-770.

(35) Sorensen TI, Sonne-Holm S. Risk in childhood of development of severe adult obesity: retrospective, population-based case-cohort study. *Am J Epidemiol* 1988; 127(1):104-113.

(36) Baker JL, Sorensen TI. The Copenhagen School Health Records Register. *Scand J Public Health* 2011; 39(7 Suppl):87-90.

(37) Baker JL, Sorensen TI. Obesity research based on the Copenhagen School Health Records Register. *Scand J Public Health* 2011; 39(7 Suppl):196-200.

(38) Goldstein H. Multilevel statistical models. 2nd edition ed. London: Edward Arnold; 1995.

(39) Tilling K, Davies NM, Nicoli E, et al. Associations of growth trajectories in infancy and early childhood with later childhood outcomes. *Am J Clin Nutr* 2011.

(40) MLwiN Version 2.26. Centre for Multilevel Modelling, University of Bristol [2012.

(41) Stata 12.0 [Texas: StataCorp; 2011.

(42) runmlwin: Stata module for fitting multilevel models in the MLwiN software. Centre for Multilevel Modelling, University of Bristol [2011.

(43) Wills AK, Lawlor DA, Matthews FE, et al. Life course trajectories of systolic blood pressure using longitudinal data from eight UK cohorts. *PLoS Med* 2011; 8(6):e1000440.

(44) Tobin MD, Sheehan NA, Scurrah KJ, et al. Adjusting for treatment effects in studies of quantitative traits: antihypertensive therapy and systolic blood pressure. *Stat Med* 2005; 24(19):2911-2935.

(45) Wu J, Kraja AT, Oberman A, et al. A summary of the effects of antihypertensive medications on measured blood pressure. *Am J Hypertens* 2005; 18(7):935-942.

(46) Fang J, Madhaven S, Alderman MH. Pulse pressure: a predictor of cardiovascular mortality among young normotensive subjects. *Blood Pressure* 2000; 9:260-266.

(47) Zimmermann E, Holst C, Sorensen TI. Lifelong doubling of mortality in men entering adult life as obese. *Int J Obes (Lond)* 2011; 35(9):1193-1199.

(48) Zimmermann E, Holst C, Sorensen TI. Morbidity, including fatal morbidity, throughout life in men entering adult life as obese. *PLoS One* 2011; 6(4):e18546.

For peer review only - http://bmjopen.bmj.com/site/about/guidelines.xhtml

(49) Black E, Holst C, Astrup A, et al Long-term influences of body-weight changes, independent of the attained weight, on risk of impaired glucose tolerance and Type 2 diabetes. *Diabet Med* 2005; 22(9):1199-1205.

(50) Wildman R, Muntner P, Reynolds K, et al.. The obese without cardiometabolic risk factor clustering and the normal weight with cardiometabolic risk factor clustering: prevalence and correlates of 2 phenotypes among the US population (NHANES 1999-2004) . *Arch Intern Med* 2008; 168:1617-1624.

(51) Gordon-Larsen P, Adair LS, Meigs JB, et al.. Discordant risk: Overweight and cardiometabolic risk in chinese adults. *Obesity (Silver Spring)* 2013; 21(1):E166-E174.

(52) Lawlor DA, Benfield L, Logue J, et al. Association between general and central adiposity in childhood, and change in these, with cardiovascular risk factors in adolescence: prospective cohort study. *BMJ* 2010; 341:c6224.

(53) Statland B, Winkel P, Bokelund H. Factors contributing to intra-individual variation of serum constituents: 2. Effects of exercise and diet on variation of serum constituents in healthy subjects. *Clinical Chemistry* 1973; 19:1380-1383.

For peer review only

Do rapid BMI growth in childhood and early-onset obesity offer cardiometabolic protection to obese adults in mid-life? Analysis of a ~~prospective~~ longitudinal cohort study of Danish men

Laura D Howe*[1], Esther Zimmermann[2], Ram Weiss[3], Thorkild IA Sørensen[2,4]

1 – MRC Integrative Epidemiology Unit at the University of Bristol, School of Social and Community Medicine, University of Bristol, Bristol, UK
2 – Institute of Preventive Medicine, Frederiksberg and Bispebjerg Hospitals, The Capital Region, Copenhagen, Denmark
3 – Braun School of Public Health, The Hebrew University of Jerusalem, Israel
4 - Novo Nordisk Center for Basic Metabolic Research, Faculty of Health and Medical Sciences, University of Copenhagen, Denmark

Key words: cardiovascular diseases, growth, metabolic syndrome, obesity, obese but metabolically healthy, metabolically abnormal but normal weight

*Corresponding author: Oakfield House, Oakfield Grove, Bristol, BS8 2BN, UK. Phone: +44 (0)117 3310134. Email: laura.howe@bristol.ac.uk

Article word length: 3,439~~3,140~~

For peer review only

Abstract

~~Background~~Objectives: Some obese individuals have no cardiometabolic abnormalities; they are 'metabolically healthy, but obese' (MHO). Similarly, some non-obese individuals have cardiometabolic abnormalities, i.e. 'metabolically at risk, normal weight' (MANW). Previous studies have suggested early-onset obesity may be associated with MHO. We aimed to assess whether BMI in childhood and early-onset obesity are associated with MHO.

~~**Design:** general population prospective cohort study~~

Setting: General population longitudinal cohort study, Denmark

~~**Objective:** To assess whether BMI in childhood and early-onset obesity are associated with MHO.~~

Participants: From 362,200 young men (mean age 20) examined for Danish National Service between 1943-1977, all obese men (BMI≥31 kg/m^2, N=1,930) were identified along with a random 1% sample of the others (N=3,601). Our analysis includes 2,392 of these men attending a research clinic in mid-life (mean age 42). For 613 of these men, data on childhood BMI is available. ~~**Deisgn:**~~ We summarised childhood BMI growth (7-13 years) using a multilevel model. Early-onset obesity was defined as obesity at examination for National Service.

Outcome measurement: We defined metabolic health at the mid-life clinic as non-fasting serum cholesterol <6.6mmol/l, non-fasting glucose <8.39mmol/l and pulse pressure <48mmHg. Participants were categorised into four groups according to their obesity (BMI≥30 kg/m^2) and metabolic health in mid-life.

Results:

297 of 1,097 (27.1%) of obese men were metabolically healthy; 826 of 1,295 (63.8%) non-obese mean had at least one metabolic abnormality. There was no evidence that rapid BMI growth in childhood or early-onset obesity was associated with either MHO or the MANW phenotype, e.g. amongst obese men in mid-life, the odds ratio for MHO comparing early-onset obesity with non-early-onset obesity was 0.97 (95% confidence interval 0.85 to 1.10).

Conclusions: We found no robust evidence that early onset obesity or rapid BMI growth in childhood is protective for cardiometabolic health.

Key words: metabolically healthy but obese, metabolic risk, cardiovascular health, obesity, child growth

For peer review only - http://bmjopen.bmj.com/site/about/guidelines.xhtml

Article summary: strengths and limitations of this study

1. This is the largest study to date to examine the question of whether childhood BMI and early onset obesity offer cardiometabolic protection to people who are obese as adults.
2. Using non-fasting measures of cholesterol and glucose and pulse pressure, we categorised men in mid life as either metabolically healthy, but obese (MHO), metabolically abnormal, obese (MAO), metabolically healthy, normal weight (MHNW) or metabolically abnormal, normal weight (MANW), and examined the relationships of childhood BMI growth and early onset obesity with this phenotype.
3. A limitation of this study is that our measures of cholesterol and glucose were from non-fasting blood samples. There is therefore potential misclassification in our measure of 'metabolically healthy'. We have tried to address this difficulty by using thresholds defined by percentiles of our data, selecting a relatively high cut-off for glucose in order to have reasonable specificity and sensitivity. Given that our estimated prevalence of MHO is within the range seen in previous studies, we feel that this is unlikely to have strongly biased our results.
4. We found no consistent evidence that rapid BMI growth in childhood, or early onset obesity, is associated with a favourable cardiometabolic profile, contradicting previous smaller studies that have examined this question.
5. Our findings imply that the notion of greater severity and metabolic impact of weight gain during adulthood is doubtful and that prevention measures should be implemented in the early childhood years.

For peer review only - http://bmjopen.bmj.com/site/about/guidelines.xhtml

Background:

Recent studies, including reviews[1-3], have drawn attention to the phenotype ‘metabolically healthy, but obese’ (MHO).[4;5] MHO individuals are defined as those who are obese but have a ‘healthy’ cardiometabolic profile; precise definitions have varied between studies, but tend to include one or more of insulin resistance, lipids, blood pressure, and markers of inflammation. The prevalence of MHO is estimated to be between 10-40% of obese people (with the remainder classified as metabolically at risk, obese, MAO).[1;6-9] Likewise, non-obese populations can be classified as either ‘metabolically healthy, normal weight’ (MHNW) or ‘metabolically at risk, normal weight’ (MANW).

Some studies have shown that MHO is associated with a reduced risk of mortality, cardiovascular disease events, type II diabetes, or a lower carotid intima-media thickness compared with MAO.[10-14] However, o~~O~~ther studies, ~~however~~including a recent systematic review[15], have demonstrated similar or greater rates of mortality[15], or development of hypertension[16-18] and type II diabetes[17;18] in MHO compared with MAO or MHNW individuals, or high rates of conversion of MHO individuals to an MAO state[19;20], suggesting that MHO is an intermediate step in the development of MAO rather than a static condition, and that no level of obesity is healthy.[15;21;22]

Several factors have been shown to be positively associated with MHO, e.g. female gender, higher physical activity (though not in all studies[23]), greater fitness[24], longer sleep duration[23], and a favourable psychosocial profile.[1] There is also some evidence that childhood growth is associated with MHO; one small study (N=117 obese children, mean age 10.4 years) found higher birth weight and faster weight gain in infancy was associated with greater levels of circulating insulin and insulin resistance, whereas weight gain after age 4 years was negatively associated with insulin resistance.[25] Analyses of 499 obese adults in Helsinki, Finland, found that those who had the metabolic syndrome were similar in birth size compared with obese people who did not have metabolic syndrome, but were lighter and thinner between 2 and 11 years of age.[26] A further study of 43 postmenopausal women observed that an earlier age at onset of obesity is more common in MHO, with 48% of MHO women reporting an age of obesity onset <20 years compared with 29% of MAO women.[27] These findings are intriguing, given the well-established association between elevated BMI in childhood and greater risk of coronary heart disease in adulthood.[28;29] Research examining the influence of growth in childhood or early-onset obesity and MHO is, however, rare, since few studies have detailed data on both childhood growth and cardiometabolic health in adulthood.

For peer review only - http://bmjopen.bmj.com/site/about/guidelines.xhtml

In this paper, we take a cohort of men from Denmark, for whom data are available on childhood growth, early onset obesity, and cardiometabolic risk factors measured in mid-life. We examine the associations of early-onset obesity and trajectories of BMI growth in childhood (from seven to 13 years) with MHO, MAO, MHNW and MANW.

Materials and Methods:

Participants, exposure measurements and sample definition:

National service is compulsory in Denmark, and before entry into service, draftees attend a physical examination where their fitness to serve is assessed. In the 1970s, a study was initiated to make use of the anthropometric data collected at these examinations. From the 362,200 men examined in the metropolitan area of Copenhagen between 1943-1977, all 1,930 men who were obese (defined as 35% overweight relative to a Scandinavian standard in use at the time, equivalent to a body mass index (BMI) ≥31kg/m^2) and a random sample of 1% of the others (N=3,601) were selected to form a cohort study. Only 3% of men eligible for national service were not examined for medical reasons, with obesity not being a condition that allowed exemption.[30] A further 2% of men were not examined because they had volunteered for service before the age of 18 years. Height was measured without shoes, and weight in only underwear. The mean age at examination was 20 years, range 18-31.

Two re-examinations were undertaken of this cohort, firstly in 1982-1984 (between 4-40 years after the draft board examination, mean age 35 years, range 22-64) and secondly in 1991-1994 (mean age of participants 45.6 years, range 33-75). All of the cohort members who were obese at draft board examination and (for logistical reasons) half of the controls were invited to these clinics, which were attended by 2,075 and 1,703 participants respectively. Previous analyses of this cohort have shown that lower BMI and higher intelligence, educational level, social class and age were associated with a greater probability of attending follow-up clinics.[31] Further details of this cohort are published elsewhere.[30;32-35] As part of a separate study[36;37], anthropometric data extracted from school health records between ages 7 and 13 are available for a subset of individuals. Participants gave informed consent, and the protocol was consistent with the principles of the Declaration of Helsinki.

For our main analysis (association between early onset obesity and obesity-metabolic status in mid-life), we included all participants who attended either the first or second follow-up clinic and had their BMI, systolic and diastolic blood pressure (SBP/DBP), cholesterol and glucose measured. We

For peer review only - http://bmjopen.bmj.com/site/about/guidelines.xhtml

used data from the second follow-up clinic where available, or the first follow-up clinic for participants attending the first but not second assessment. For our secondary analysis of the association between childhood growth trajectories and obesity-metabolic status in mid-life, this sample was further restricted to participants with one or more BMI measure from school health records between 7-13 years.

Exposure measurements:

Obesity status (BMI ≥31kg/m^2) in early adulthood (at entry into National Service, mean age 20 years) determines our measurement of early-onset obesity.

We used a multilevel model to estimate BMI at age 7 and the linear change in BMI between 7-13 years for all participants with one or more BMI measure. Multilevel models are an appropriate tool for the analysis of longitudinal data, since they allow for the non-independence of repeated measures on the same individual, and can estimate a full trajectory for all participants with one or more measurement under a missing at random assumption.[38;39] The multilevel model was run in MLwiN version 2.26[40] via Stata version 12[41], using the command runmlwin.[42] Full details of the model are provided in online supplementary material. From the multilevel model, we derived predicted BMI at age 7 and predicted linear change in BMI between 7-13 years; these variables were used as exposures in our analyses.

Outcome measurements:

At each follow-up clinic, height was measured without shoes and weight was measured in light clothes and without shoes. SBP and DBP were measured three times with the participant at rest; the mean of the 3 measures was used. Total serum glucose and cholesterol were assessed from non-fasting blood samples (examinations were undertaken during day time, and the participants were not requested to skip breakfast or lunch) drawn from a vein after light stasis. Samples were placed in a box containing ice water, and within two hours centrifuged at 3,000rpm for ten minutes. The plasma was stored at 4°C and analysed the following day. Cholesterol was measured enzymatically using the CHOD-PAP method at the first examination and high-performance CHOD-PAP at the second (Boehringer Mannheim). Glucose was assayed using the hexokinase/G6P-DH assy. Blood samples remained at room temperature for 20-60 minutes, after which the whole blood from the EDTA tube was placed in a plastic tube and frozen at -20°C. To control for treatment effects, a constant of 10mmHg was added to SBP and 5mmHg to DBP for individuals reporting treatment with antihypertensive medication (n=156); these values are consistent with the effects of antihypertensive medications, and this method has been shown to be a reasonable approach for

For peer review only - http://bmjopen.bmj.com/site/about/guidelines.xhtml

reducing treatment bias and has been used in previous studies.[43-45] Pulse pressure, identified in previous research as an important independent risk factor for cardiovascular disease[46], was defined as SBP-DBP.

For each cardiometabolic risk factor (SBP, DBP, pulse pressure, glucose and cholesterol), a measure of the level of the risk factor given what would be predicted from age and BMI was created by regressing the risk factor on age and BMI at the time of assessment, and standardising the resulting residuals to have a mean of zero and variance of one. Thus for these measures a value of zero would mean that the level of the risk factor is what would be predicted given age and BMI, positive values would mean higher than expected levels, and vice versa for negative values.

Various definitions of MHO have been used in previous studies.[1;2] Since the blood samples in our study were non-fasting, standard cut-offs based on fasting samples would be too low. We therefore opted to define cut-offs for cholesterol and glucose based on percentiles of the study-population distribution. For cholesterol, we deemed levels above the 75th centile (6.6mmol/l) to be high. Fluctuations in glucose are greater than those in cholesterol, so a higher threshold is necessary in order to obtain a measure that is reasonably sensitive but also has sufficient specificity. Therefore we defined high glucose levels as above the 95% centile of non-obese participants (8.39mmol/l). In line with previous research[46], we defined high pulse pressure as above 48. 'Metabolically healthy' was defined as having cholesterol, glucose and pulse pressure levels below these thresholds; participants with one or more measurement above these thresholds were deemed 'metabolically at risk'. Individuals were classified according to their obesity status in mid-life (obesity defined as ≥30 kg/m^2) and metabolic status, such that individuals were MHO, MAO, MHNW or MANW. As sensitivity analyses, we repeated our analyses using lower cut-points for glucose (≤5.6mmol/l) and cholesterol (<5.2mmol/l), and using systolic and diastolic blood pressure instead of pulse pressure.

Other data:

The occupation of participants' fathers was self-reported in questionnaires and coded as manual or non-manual. A binary indicator of participants' smoking behaviour (any versus no smoking) was created from self-reported data.

Statistical analysis:

The associations of early onset obesity and childhood growth with the cardiometabolic risk factors and standardised residuals from regression of cardiometabolic risk factors on age and BMI were

For peer review only - http://bmjopen.bmj.com/site/about/guidelines.xhtml

assessed using linear regression. The associations of early onset obesity and childhood growth with MHO (compared with MAO) or with MHNW (compared with MANW) were assessed using logistic regression. As a sensitivity analysis, we assessed whether our findings are robust to adjustment for father's social class and participants' smoking status.

Results:

2,392 participants were included in our analyses; of these, data from the second follow-up assessment were available for 1,691 and from the first assessment for a further 701 (**Table 1**).

Prevalence of obesity and metabolic phenotypes:

Amongst the 2,392 participants, 1,295 were not obese. Of these, 469 (36.2%) were classified as MHNW, and 826 (63.8%) as MANW (**Table 1**). Thus 63.8% of normal weight individuals had one or more metabolic abnormality. Of the 1,097 participants who were obese at follow-up, 297 (27.1%) were MHO.

Compared with MANW individuals, MHNW individuals tended to be younger (mean and standard deviation (SD) for age 44.93 (10.89) and 42.09 (8.40) years respectively) and thinner (mean and SD for BMI 25.55 (2.69) and 24.82 (2.85) years) (**Table 2**). MAO had higher BMI compared with MHO individuals (mean and SD BMI 36.49 (5.10) and 35.79 (4.53) kg/m^2), but there was little difference in age (**Table 2**).

Childhood growth trajectories:

Differences in BMI between participants who were obese and non-obese at draft board examination were already evident by age 7. The mean BMI at age 7 and rate of BMI change between 7-13 years were higher in those who were obese at draft board (**Web Table 1**). The multilevel model had good fit to the data (**Web Table 2** and **Web Figures 1-3**).

Associations of childhood growth and early onset obesity with cardiometabolic health and obesity/metabolic phenotypes in mid-life:

Those with early onset obesity (i.e. those who were obese at draft board examination) had a BMI in mid-life on average 9.65 kg/m^2 (95% CI 9.27 to 10.03) greater than those who were not obese at the draft board examination. Greater BMI at age 7, faster rate of BMI increase between 7-13 years and

For peer review only - http://bmjopen.bmj.com/site/about/guidelines.xhtml

early onset obesity were all associated with greater SBP, DBP and pulse pressure, and greater glucose levels in mid-life (**Table 3**). These associations tended to increase in magnitude after adjustment for age at the research clinic and year of draft board examination. In our analyses, tThe associations of childhood growth and early onset obesity with levels of cholesterol were weaker than for the other cardiometabolic risk factors.

There was very little evidence of associations of childhood growth and early onset obesity with standardised scores of the cardiometabolic risk factors that account for age and BMI at the time of assessment (**Table 4**). There was no evidence of associations for the standardised scores of SBP or DBP. For standardised scores of glucose, early onset obesity and faster rate of BMI increase between 7-13 years were both associated with higher scores (no association was seen for BMI at 7 years). For example, after adjustment for year at draft board, early onset obesity was associated with a 0.16 SD higher standardised score of glucose (95% CI 0.08 to 0.24), and a 1 kg/m^2 per year increase in the rate of BMI growth between 7-13 years (equivalent to approximately 2 SD of the rate of growth) was associated with a 0.26 SD higher standardised score (95% CI 0.03 to 0.49). Conversely, early onset obesity, greater BMI at age 7 and faster rate of BMI increase between 7-13 years were all associated with lower standardised scores of cholesterol. For example, after adjustment for year of draft board examination, early onset obesity was associated with a -0.19 lower standardised score for cholesterol (95% CI -0.27 to -0.11) and a 1 kg/m^2 per year increase in the rate of BMI growth between 7-13 years was associated with a -0.12 lower standardised score (95% CI -0.35 to 0.10). Thus early onset obesity and faster rates of BMI increase in childhood are associated with a higher glucose level but lower cholesterol level than would be predicted from age and adult BMI.

Among those who were not obese at the follow-up clinic, early onset obesity was associated with slightly lower odds of being MHNW compared with MANW, but the confidence interval was wide and included the null value (odds ratio 0.76, 95% CI 0.54 to 1.07) (**Table 5**). There was no consistent relationship between childhood BMI trajectories and MHNW compared with MANW.

Among those who were obese in mid-life, there was a weak association between early onset obesity and lower odds of being MHO compared with MAO; odds ratio 0.69, 95% CI 0.46 to 1.04. There was no association between BMI at age 7 or BMI increases between 7-13 years and MHO (**Table 5**). Results were unchanged when adjusted for father's social class and participant's smoking (**Web Table 3**).

For peer review only - http://bmjopen.bmj.com/site/about/guidelines.xhtml

Associations of early onset obesity and child growth with MHO remained similar if lower thresholds for defining abnormal cholesterol and glucose were used, or if systolic and diastolic blood pressure were used in the definition of metabolically healthy instead of pulse pressure (results available from authors on request).

Discussion:

We found little consistent evidence to support the hypothesis that rapid BMI growth in childhoodbetween 7-13 years and early onset obesity are associated with a protective cardiometabolic effect. Early onset obesity and faster rates of BMI increase in childhood were associated with a higher glucose level but lower cholesterol level than would be predicted from age and adult BMI. No associations were seen for SBP or DBP levels or pulse pressure adjusted for concurrent age and BMI. There was also no consistent evidence that early onset obesity or rates of BMI change in childhood were associated with being 'metabolically healthy' – early onset obesity was weakly associated with lower odds of being MHO, but no associations were seem for BMI at age 7 or rate of BMI change between 7-13 years. Our findings contrast with a study of 43 postmenopausal women, which observed that an earlier age at onset of obesity is more common in MHO, with 48% of MHO women reporting reported an age of obesity onset <20 years compared with 29% of MAO women.[27] One small previous study of 117 obese children found that faster weight gain in infancy with greater levels of circulating insulin and reduced insulin resistance, whereas weight gain after age 4 years was associated with higher insulin resistance. The earliest available childhood BMI measures in our cohort are from age 7; thus it is possible that BMI changes at earlier stages could be associated with being metabolically healthy, and we are thus unable to make direct comparisons between our results and those of the studies looking at birth size and weight gain in early childhood in relation to MHO.[25;26] Our findings are supported by some previous research on this cohort, which has found that the men who were obese at their draft board examination in early adulthood experience a doubling of all-cause mortality risk[47], as well as increased risks of mortality from cardiovascular diseases and a wide range of other specific causes.[48] However, other analyses of this cohort have demonstrated that obese men, maintaining their weight since the draft board examination in early adulthood, had lower risk of impaired glucose tolerance than non-obese men in early adulthood who became similarly obese by age 51.[49]

For peer review only - http://bmjopen.bmj.com/site/about/guidelines.xhtml

In this cohort of Danish men, 27.1% of participants who were obese at follow-up were classified as MHO defined by having non-fasting cholesterol <6.6mmol/l, non-fasting glucose <8.39mmol/l and pulse pressure <48mmHg. This is within the range of 10-40% prevalence reported in previous studies.[1;6-9] A large study of over 5000 adults in the National Health and Nutrition Examination Surveys from the USA found a prevalence of MHO of 51.3%.[50] However, this study differed from ours in two important ways; firstly by considering overweight participants rather than obese, and secondly by considering metabolically at risk to be two or more abnormalities, whereas we assumed one or more abnormality conferred risk.

Notably, a very high proportion of non-obese participants were classified as MANW: 63.8%. Analysis of data from a Chinese cohort also found a high prevalence of cardiometabolic abnormalities in non-obese adults[51], leading the authors to conclude that focusing cardiovascular screening only on obese individuals would miss a large proportion of the at risk population. The NHANES data also found that 23.5% of non-overweight participants had at least two metabolic abnormalities.[50] In our study, the mean BMI in the non-obese group was high – 24.82 kg/m^2 in the MHNW group and 25.55 in the MANW group. Thus although not obese, many of these participants were overweight.

Our analysis included 2,392 individuals, 1,097 of whom were obese, making it larger than most existing studies on MHO.[1;2] We were only able to include a subset of these individuals (N=613) in our analysis of the association between childhood BMI growth and later metabolic health. This is a larger sample size than the former studies on this topic[25;27], but we may still have had limited power to detect an association. We also were unable to examine the influence of growth before age 7 or of puberty. Furthermore, some of our participants were quite young at the follow-up examinations, and so associations may emerge in later life as cardiovascular risk develops. Our measure of early-onset obesity is based on obesity at a mean age of 20 years; whilst we feel that this is an appropriate definition of early-onset obesity in this population, who grew up at a time when childhood obesity was very rare, studies in younger cohorts may wish to consider earlier ages to define 'early' obesity. Our measures of adiposity are based on BMI, which is an imperfect measure of fatness, particularly in children. However, even in childhood, BMI has been shown to have similar associations with cardiovascular risk factors as directly-determined fat mass.[52]

We did not have data on insulin or on lipids other than total cholesterol, or other measures that may be stronger predictors of cardiovascular events, and therefore we could not incorporate these into our definitions of MHO, MAO, MHNW and MANW. Our measures of cholesterol and glucose were

For peer review only - http://bmjopen.bmj.com/site/about/guidelines.xhtml

from non-fasting blood samples. There is therefore potential misclassification in our measure of 'metabolically healthy'. We have tried to address this difficulty by using thresholds defined by percentiles of our data. Glucose levels are likely to fluctuate more in non-fasting samples than cholesterol levels[53], and we therefore selected a relatively high cut-off for glucose in order to have reasonable specificity and sensitivity. Given that our estimated prevalence of MHO is within the range seen in previous studies, we feel that this is unlikely to have strongly biased our results, which remained similar if lower cut-points were used for cholesterol and glucose.

We found no consistent evidence that rapid BMI growth in childhood, or early onset obesity, is associated with a favourable cardiometabolic profile, contradicting previous smaller studies that have examined this question. Our findings imply that the notion of greater severity and metabolic impact of weight gain during adulthood is doubtful and that prevention measures should be implemented in the early childhood years.

For peer review only

For peer review only - http://bmjopen.bmj.com/site/about/guidelines.xhtml

Contributions of each author: The study was conceived by LDH and TIAS; the study was designed by LDH with input from TIAS and RW; LDH carried out statistical analyses and wrote the first draft of the manuscript; all authors contributed to critical revisions of the manuscript and approved the final submitted version

Data sharing statement: statistical code can be requested from the corresponding author. For details of data access and collaboration policy, please contact Thokild Sørensen: tias@sund.ku.dk

Funding statement: This work was supported by a UK Medical Research Fellowship to LDH (G1002375); the funders had no role in the design or conduct of the study or the decision to publish.

Competing interests statement: Competing interests: None declared

For peer review only

Table 1: Sample characteristics, mean (SD) or % characteristics of participants included in analyses

	Participants included in analysis of the association between early-onset obesity and metabolic health in mid-life			Participants included in analysis of the association between childhood growth and metabolic health in mid-life		
	Participants attending second follow-up in adulthood	Participants attending first follow-up in adulthood but not the second follow-up	Combined whole sample	Participants attending second follow-up in adulthood	Participants attending first follow-up in adulthood but not the second follow-up	Combined whole sample
	**N=1,691**	**N=701**	**N=2,392**	**N=420**	**N=193**	**N=613**
Age (years)	45.61 (7.93)	34.81 (8.38)	42.44 (9.45)	48.01 (6.82)	36.34 (6.90)	44.34 (8.73)
Number (%) obese in early adulthood*	784 (46.4%)	346 (49.4%)	1,130 (47.2%)	189 (45.0%)	95 (49.2%)	284 (46.3%)
Body mass index (kg/m^2)	30.57 (6.69)	29.76 (6.87)	30.34 (6.75)	30.52 (6.79)	31.00 (7.74)	30.67 (7.10)
Systolic blood pressure (mmHg)	142.37 (18.12)	138.50 (16.18)	141.89 (18.32)	143.64 (18.75)	138.08 (16.33)	141.89 (18.19)
Diastolic blood pressure (mmHg)	92.81 (11.41)	86.45 (12.25)	91.27 (12.31)	92.29 (10.81)	89.27 (11.70)	91.34 (11.18)
Pulse pressure (mmHg)	49.96 (13.26)	52.22 (12.38)	50.62 (13.04)	51.36 (13.73)	48.80 (11.91)	50.55 (13.23)
Glucose (mmol/l)	6.48 (2.95)	6.57 (2.42)	6.51 (2.80)	6.56 (3.14)	6.65 (2.52)	6.59 (2.96)
Cholesterol (mmol/l)	6.10 (1.28)	5.25 (1.22)	5.85 (1.32)	6.11 (1.20)	5.34 (1.24)	6.86 (1.26)
*Number (%) with metabolic phenotypes ** *						
NON-OBESE AT MID-LIFE FOLLOW-UP	*N=903*	*N=392*	*N=1,295*	*N=235*	*N=102*	*N=337*
Metabolically healthy	328 (36.3%)	141 (36.0%)	469 (36.2%)	81 (34.5%)	49 (48.0%)	130 (38.6%)
Metabolically at risk	575 (63.7%)	251 (64.0%)	826 (63.8%)	154 (65.5%)	53 (52.0%)	207 (61.4%)
OBESE AT MID-LIFE FOLLOW-UP	*N=788*	*N=309*	*N=1,097*	*N=185*	*N=91*	*N=276*
Metabolically healthy, but obese	221 (28.0%)	76 (24.6%)	297 (27.1%)	48 (25.9%)	28 (30.8%)	76 (27.5%)
Metabolically at risk, obese	567 (72.0%)	233 (75.4%)	800 (72.9%)	137 (74.1%)	63 (69.2%)	200 (72.5%)

*Defined as 35% overweight relative to a Scandinavian standard in use at the time, equivalent to a body mass index (BMI) ≥31kg/m^2

**Metabolically healthy is defined by having pulse pressure (systolic – diastolic blood pressure) <48mmHg, glucose <8.39mmol/l, and cholesterol <6.6mmol/l; i.e. any one or more metabolic abnormality confers 'metabolically at-risk' status. Obesity at the mid-life follow-up is defined by BMI≥30kg/m^2

For peer review only - http://bmjopen.bmj.com/site/about/guidelines.xhtml

Table 2: Characteristics of participants within each metabolic and obesity category

	Metabolically healthy, normal weight (MHNW)*	Metabolically at risk, normal weight (MANW)*	Metabolically healthy, but obese (MHO)*	Metabolically at risk, obese (MAO)*
Mean (SD)				
	N=469	N=826	N=297	N=800
Age (years)	42.09 (8.40)	44.93 (10.89)	40.15 (7.33)	40.93 (8.53)
BMI (kg/m^2)	24.82 (2.85)	25.55 (2.69)	35.79 (4.53)	36.49 (5.10)
Systolic blood pressure (mmHg)	126.60 (10.37)	142.97 (16.98)	134.70 (10.74)	152.41 (18.22)
Diastolic blood pressure (mmHg)	86.85 (9.21)	88.00 (12.34)	95.21 (9.77)	95.77 (12.76)
Pulse pressure (mmHg)	39.74 (5.23)	54.97 (11.97)	39.49 (5.67)	56.64 (12.73)
Cholesterol (mmol/l)	5.26 (0.87)	6.06 (1.40)	5.39 (0.82)	6.15 (1.45)
Glucose (mmol/l)	5.62 (0.87)	6.29 (2.30)	5.76 (0.74)	7.53 (3.95)
Number (%) that were obese in early adulthood**	61 (13.0%)	118 (14.3%)	253 (85.2%)	698 (87.3%)

* Metabolically healthy is defined by having pulse pressure (systolic – diastolic blood pressure) <48mmHg, glucose <8.39mmol/l, and cholesterol <6.6mmol/l; i.e. any one or more metabolic abnormality confers 'metabolically at-risk' status. Obesity in mid-life is defined by BMI≥30kg/m^2

**Obesity in early adulthood is defined as 35% overweight relative to a Scandinavian standard in use at the time, equivalent to a body mass index (BMI) ≥31kg/m^2

For peer review only - http://bmjopen.bmj.com/site/about/guidelines.xhtml

Table 3: Linear regression coefficients (with 95% confidence intervals) for the association of early onset obesity and childhood BMI trajectories with cardiovascular risk factors measured in mid-life.

Model*	Exposure	Outcome					
		BMI (kg/m^2)	Systolic blood pressure (mmHg)	Diastolic blood pressure (mmHg)	Pulse pressure (mmHg)	Glucose (mmol/l)	Cholesterol (mmol/l)
Full sample, N=2,329							
1	Early onset obesity**	9.65 (9.27 to 10.03) p<0.001	7.70 (6.27 to 9.14) p<0.001	5.31 (4.35 to 6.28) p<0.001	2.39 (1.35 to 3.44) p<0.001	1.14 (0.92 to 1.36) p<0.001	-0.14 (-0.25 to -0.04) p=0.01
2		9.76 (9.36 to 10.16) p<0.001	10.28 (8.82 to 11.74) p<0.001	6.02 (5.01 to 7.02) p<0.001	4.27 (3.21 to 5.33) p<0.001	1.30 (1.07 to 1.53) p<0.001	-0.07 (-0.18 to 0.04) p=0.19
Subsample with childhood growth data, N=613							
1	BMI at 7 years (kg/m^2)	1.81 (1.60 to 2.02) p<0.001	1.37 (0.72 to 2.02) p<0.001	0.72 (0.32 to 1.12) p<0.001	0.65 (0.17 to 1.13) p=0.01	0.12 (0.02 to 0.23) p=0.03	-0.04 (-0.09 to 0.00) p=0.08
2		1.75 (1.53 to 1.97) p<0.001	1.82 (1.17 to 2.47) p<0.001	0.85 (0.44 to 1.26) p<0.001	0.97 (0.49 to 1.45) p<0.001	0.13 (0.02 to 0.24) p=0.02	-0.02 (-0.06 to 0.03) p=0.50
1	BMI change 7-13 years (kg/m^2 per year)	10.95 (10.01 to 11.89) p<0.001	8.47 (5.26 to 11.67) p<0.001	4.53 (2.55 to 6.51) p<0.001	3.93 (1.57 to 6.30) p=0.001	1.31 (0.78 to 1.83) p<0.001	-0.20 (-0.42 to 0.03) p=0.09
2		8.75 (7.59 to 9.91) p<0.001	9.69 (5.76 to 13.63) p<0.001	4.76 (2.27 to 7.25) p<0.001	4.93 (2.00 to 7.87) p=0.001	1.58 (0.91 to 2.24) p<0.001	0.05 (-0.22 to 0.33) p=0.71

*Model details: Model 1 is unadjusted; Model 2 is adjusted for age at outcome assessment and year of draft board examination, and the coefficient for change in BMI between 7-11 years is additionally adjusted for BMI at 7 years as predicted by the multilevel model

**Defined as 35% overweight relative to a Scandinavian standard in use at the time, equivalent to a body mass index (BMI) ≥31kg/m^2 in early adulthood, mean age 20 years

For peer review only

Table 4: Linear regression coefficients (with 95% confidence intervals) for the association of early onset obesity and childhood BMI trajectories with levels of cardiovascular risk factors in mid-life adjusted for age and BMI in mid-life.

Model*	Exposure	Outcomes** (standard deviations, given age and concurrent BMI)				
		Systolic blood pressure	Diastolic blood pressure	Pulse pressure	Glucose	Cholesterol
Full sample, N=2,329						
1	Early onset obesity***	0.02 (-0.06 to 0.10) p=0.62	-0.01 (-0.09 to 0.07) p=0.78	0.04 (-0.04 to 0.12) p=0.38	0.13 (0.05 to 0.21) p=0.002	-0.13 (-0.21 to -0.05) p=0.002
2		0.05 (-0.04 to 0.13) p=0.28	-0.06 (-0.14 to 0.03) p=0.18	0.11 (0.03 to 0.19) p=0.01	0.16 (0.08 to 0.24) p<0.001	-0.19 (-0.27 to -0.11) p<0.001
Subsample with childhood growth data, N=613						
1	BMI at 7 years (kg/m^2)	0.00 (-0.04 to 0.04) p=0.97	-0.01 (-0.05 to 0.03) p=0.57	0.01 (-0.03 to 0.05) p=0.61	-0.01 (-0.05 to 0.02) p=0.42	-0.03 (-0.07 to 0.00) p=0.07
2		0.00 (-0.03 to 0.04) p=0.89	-0.01 (-0.05 to 0.02) p=0.47	0.01 (-0.02 to 0.05) p=0.49	-0.01 (-0.05 to 0.02) p=0.50	-0.04 (-0.08 to -0.01) p=0.02
1	BMI change 7-13 years (kg/m^2 per year)	0.03 (-0.15 to 0.21) p=0.75	-0.04 (-0.22 to 0.14) p=0.69	0.07 (-0.11 to 0.25) p=0.46	0.11 (-0.07 to 0.29) p=0.24	-0.15 (-0.33 to 0.03) p=0.10
2		0.05 (-0.18 to 0.28) p=0.65	-0.02 (-0.25 to 0.20) p=0.83	0.07 (-0.16 to 0.31) p=0.52	0.26 (0.03 to 0.49) p=0.03	-0.12 (-0.35 to 0.10) p=0.28

The outcome in these analyses is a measure of the level of the cardiovascular risk factor (SBP, DBP, pulse pressure, glucose and cholesterol) given what would be predicted from age and BMI; this was created by regressing the risk factor on age and BMI at the time of assessment, and standardising the resulting residuals to have a mean of zero and variance of one. Thus for these measures a value of zero would mean that the level of the risk factor is what would be predicted given age and BMI, positive values would mean higher than expected levels, and vice versa for negative values.

*Model details: Model 1 is unadjusted; Model 2 is adjusted for year of draft board examination, in model 2 the coefficient for change in BMI between 7-11 years is additionally adjusted for BMI at 7 years as predicted by the multilevel model

**Cardiovascular risk factors were regressed on concurrent BMI and age, and standardised residuals (mean of zero, variance of one) from those regressions are used as the outcomes in these analyses. Thus a positive value of these outcomes means that an individual has a higher level of that outcome than would be expected given their age and BMI, and a negative value means the opposite, lower level of the outcome given their age and BMI

***Defined as 35% overweight relative to a Scandinavian standard in use at the time, equivalent to a body mass index (BMI) ≥31kg/m^2 in early adulthood, mean age 20 years

For peer review only - http://bmjopen.bmj.com/site/about/guidelines.xhtml

Table 5: Odds ratios (with 95% confidence intervals) for the association of obese status in early adulthood and growth in early childhood with metabolic status in mid-life; stratified by mid-life obesity status

Model*	Metabolically healthy, normal weight**	Metabolically at risk, normal weight**	Metabolically healthy, but obese**	Metabolically at risk, obese**
Full sample, N=2,329				
	N=469	*N=826*	*N=297*	*N=800*
**Early onset obesity*** **				
1	0.90 (0.64 to 1.25) p=0.52	1 (ref)	0.84 (0.57 to 1.23) p=0.37	1 (ref)
2	0.76 (0.54 to 1.07) p=0.12	1 (ref)	0.69 (0.46 to 1.04) p=0.08	1 (ref)
Subsample with childhood growth data, N=613				
	N=130	*N=207*	*N=76*	*N=200*
**BMI at 7 (kg/m^2)**				
1	0.97 (0.85 to 1.10) p=0.60	1 (ref)	0.99 (0.88 to 1.13) p=0.92	1 (ref)
2	0.93 (0.81 to 1.07) p=0.32	1 (ref)	0.97 (0.85 to 1.10) p=0.65	1 (ref)
**BMI change 7-13 (kg/m^2 per year)**				
1	0.85 (0.41 to 1.70) p=0.62	1 (ref)	1.50 (0.73 to 3.05) p=0.27	1 (ref)
2	0.66 (0.25 to 1.72) p=0.39	1 (ref)	1.40 (0.61 to 3.48) p=0.38	1 (ref)

Coefficients are odds ratios (95% confidence intervals) comparing obese in early adulthood with non-obese in early adulthood, or a one unit increase in BMI at age 7 or the linear rate of change in BMI between 7 and 13 years.

*Model details: Model 1 is unadjusted; Model 2 is adjusted for age at outcome assessment and year of draft board examination. In model 2 the coefficient for change in BMI between 7-13 years is additionally adjusted for BMI at 7 years as predicted by the multilevel model

** Metabolically healthy is defined by having pulse pressure (systolic – diastolic blood pressure) <48mmHg, glucose <8.39mmol/l, and cholesterol <6.6mmol/l; i.e. any one or more metabolic abnormality confers 'metabolically at-risk' status. Obesity in mid-life is defined by BMI≥30kg/m^2

***Defined as 35% overweight relative to a Scandinavian standard in use at the time, equivalent to a body mass index (BMI) ≥31kg/m^2 in early adulthood, mean age 20 years

For peer review only

Reference List

(1) Primeau V, Coderre L, Karelis AD, Brochu M, Lavoie ME, Messier V et al. Characterizing the profile of obese patients who are metabolically healthy. *Int J Obes* 2011; 35(7):971-981.

(2) Denis GV, Obin MS. 'Metabolically healthy obesity': Origins and implications. *Mol Aspects Med* 2012.

(3) Ahima RS, Lazar MA. The health risk of obesity - better metrics imperative. *Science* 2013; 341:856.

(4) Sorensen TI, Virtue S, Vidal-Puig A. Obesity as a clinical and public health problem: is there a need for a new definition based on lipotoxicity effects? *Biochim Biophys Acta* 2010; 1801(3):400-404.

(5) Sorensen TI. Obesity defined as excess storage of inert triglycerides--do we need a paradigm shift? *Obes Facts* 2011; 4(2):91-94.

(6) Karelis AD, Brochu M, Rabasa-Lhoret R. Can we identify metabolically healthy but obese individuals (MHO)? *Diabetes Metab* 2004; 30(6):569-572.

(7) Karelis AD, Brochu M, Rabasa-Lhoret R, Garrel D, Poehlman ET. Clinical markers for the identification of metabolically healthy but obese individuals. *Diabetes Obes Metab* 2004; 6(6):456-457.

(8) Karelis AD, St-Pierre DH, Conus F, Rabasa-Lhoret R, Poehlman ET. Metabolic and body composition factors in subgroups of obesity: what do we know? *J Clin Endocrinol Metab* 2004; 89(6):2569-2575.

(9) Karelis AD. Metabolically healthy but obese individuals. *Lancet* 2008; 372(9646):1281-1283.

(10) Meigs JB, Wilson PW, Fox CS, Vasan RS, Nathan DM, Sullivan LM et al. Body mass index, metabolic syndrome, and risk of type 2 diabetes or cardiovascular disease. *J Clin Endocrinol Metab* 2006; 91(8):2906-2912.

(11) Durward CM, Hartman TJ, Nickols-Richardson SM. All-Cause Mortality Risk of Metabolically Healthy Obese Individuals in NHANES III. *J Obes* 2012; 2012:460321.

(12) Hamer M, Stamatakis E. Metabolically healthy obesity and risk of all-cause and cardiovascular disease mortality. *J Clin Endocrinol Metab* 2012; 97(7):2482-2488.

(13) Calori G, Lattuada G, Piemonti L, Garancini MP, Ragogna F, Villa M et al. Prevalence, metabolic features, and prognosis of metabolically healthy obese Italian individuals: the Cremona Study. *Diabetes Care* 2011; 34(1):210-215.

(14) Li S, Chen W, Srinivasan SR, Xu J, Berenson GS. Relation of childhood obesity/cardiometabolic phenotypes to adult cardiometabolic profile: the Bogalusa Heart Study. *Am J Epidemiol* 2012; 176 Suppl 7:S142-S149.

For peer review only

(15) Kramer CK, Zinman B, Retnakaran R. Are metabolically healthy overweight and obesity benign conditions?: A systematic review and meta-analysis. *Ann Intern Med* 2013; 159(11):758-769.

(16) Lee SK, Kim SH, Cho GY, Baik I, Lim HE, Park CG et al. Obesity phenotype and incident hypertension: a prospective community-based cohort study. *J Hypertens* 2013; 31(1):145-151.

(17) Hwang LC, Bai CH, Sun CA, Chen CJ. Prevalence of metabolically healthy obesity and its impacts on incidences of hypertension, diabetes and the metabolic syndrome in Taiwan. *Asia Pac J Clin Nutr* 2012; 21(2):227-233.

(18) Chang Y, Ryu S, Suh BS, Yun KE, Kim CW, Cho SI. Impact of BMI on the incidence of metabolic abnormalities in metabolically healthy men. *Int J Obes (Lond)* 2012; 36(9):1187-1194.

(19) Appleton SL, Seaborn CJ, Visvanathan R, Hill CL, Gill TK, Taylor AW et al. Diabetes and Cardiovascular Disease Outcomes in the Metabolically Healthy Obese Phenotype: A cohort study. *Diabetes Care* 2013.

(20) Bradshaw PT, Monda KL, Stevens J. Metabolic syndrome in healthy obese, overweight, and normal weight individuals: The atherosclerosis risk in communities study. *Obesity (Silver Spring)* 2013; 21(1):203-209.

(21) Hill JO, Wyatt HR. The myth of healthy obesity. *Ann Intern Med* 2013; 159(11):789-790.

(22) Thomsen M, Nordestgaard BG. Myocardial infarction and ischemic heart disease in overweight and obesity with and without metabolic syndrome. *JAMA Intern Med* 2014; 174(1):15-22.

(23) Hankinson AL, Daviglus ML, Van HL, Chan Q, Brown I, Holmes E et al. Diet composition and activity level of at risk and metabolically healthy obese American adults. *Obesity (Silver Spring)* 2012.

(24) Ortega FB, Lee DC, Katzmarzyk PT, Ruiz JR, Sui X, Church TS et al. The intriguing metabolically healthy but obese phenotype: cardiovascular prognosis and role of fitness. *Eur Heart J* 2013; 34(5):389-397.

(25) Bouhours-Nouet N, Dufresne S, de Casson FB, Mathieu E, Douay O, Gatelais F et al. High birth weight and early postnatal weight gain protect obese children and adolescents from truncal adiposity and insulin resistance: metabolically healthy but obese subjects? *Diabetes Care* 2008; 31(5):1031-1036.

(26) Salonen MK, Kajantie E, Osmond C, Forsen T, Yliharsila H, Paile-Hyvarinen M et al. Role of childhood growth on the risk of metabolic syndrome in obese men and women. *Diabetes Metab* 2009; 35(2):94-100.

(27) Brochu M, Tchernof A, Dionne IJ, Sites CK, Eltabbakh GH, Sims EA et al. What are the physical characteristics associated with a normal metabolic profile despite a high level of obesity in postmenopausal women? *J Clin Endocrinol Metab* 2001; 86(3):1020-1025.

(28) Baker JL, Olsen LW, Sorensen TI. Childhood body-mass index and the risk of coronary heart disease in adulthood. *New England Journal of Medicine* 2007; 357:2329-2337.

For peer review only - http://bmjopen.bmj.com/site/about/guidelines.xhtml

(29) Owen CG, Whincup PH, Orfei L, Chou Q-A, Rudnicka AR, Wathern AK et al. Is body mass index before middle age related to coronary heart disease risk in later life? Evidence from observational studies. *International Journal of Obesity* 2009; 33:866-877.

(30) Sorensen TI, Sonne-Holm S. Mortality in extremely overweight young men. *J Chronic Dis* 1977; 30(6):359-367.

(31) Sonne-Holm S, Sorensen TI, Jensen G, Schnohr P. Influence of fatness, intelligence, education and sociodemographic factors on response rate in a health survey. *J Epidemiol Community Health* 1989; 43(4):369-374.

(32) Sonne-Holm S, Sorensen TI, Christensen U. Risk of early death in extremely overweight young men. *Br Med J (Clin Res Ed)* 1983; 287(6395):795-797.

(33) Sonne-Holm S, Sorensen TI, Jensen G, Schnohr P. Long-term changes of body weight in adult obese and non-obese men. *Int J Obes* 1990; 14(4):319-326.

(34) Sonne-Holm S, Sorensen TI, Jensen G, Schnohr P. Independent effects of weight change and attained body weight on prevalence of arterial hypertension in obese and non-obese men. *BMJ* 1989; 299(6702):767-770.

(35) Sorensen TI, Sonne-Holm S. Risk in childhood of development of severe adult obesity: retrospective, population-based case-cohort study. *Am J Epidemiol* 1988; 127(1):104-113.

(36) Baker JL, Sorensen TI. The Copenhagen School Health Records Register. *Scand J Public Health* 2011; 39(7 Suppl):87-90.

(37) Baker JL, Sorensen TI. Obesity research based on the Copenhagen School Health Records Register. *Scand J Public Health* 2011; 39(7 Suppl):196-200.

(38) Goldstein H. Multilevel statistical models. 2nd edition ed. London: Edward Arnold; 1995.

(39) Tilling K, Davies NM, Nicoli E, Ben-Shlomo Y, Kramer MS, Patel R et al. Associations of growth trajectories in infancy and early childhood with later childhood outcomes. *Am J Clin Nutr* 2011.

(40) MLwiN Version 2.26. Centre for Multilevel Modelling, University of Bristol [2012.

(41) Stata 12.0 [Texas: StataCorp; 2011.

(42) runmlwin: Stata module for fitting multilevel models in the MLwiN software. Centre for Multilevel Modelling, University of Bristol [2011.

(43) Wills AK, Lawlor DA, Matthews FE, Sayer AA, Bakra E, Ben-Shlomo Y et al. Life course trajectories of systolic blood pressure using longitudinal data from eight UK cohorts. *PLoS Med* 2011; 8(6):e1000440.

(44) Tobin MD, Sheehan NA, Scurrah KJ, Burton PR. Adjusting for treatment effects in studies of quantitative traits: antihypertensive therapy and systolic blood pressure. *Stat Med* 2005; 24(19):2911-2935.

For peer review only - http://bmjopen.bmj.com/site/about/guidelines.xhtml

(45) Wu J, Kraja AT, Oberman A, Lewis CE, Ellison RC, Arnett DK et al. A summary of the effects of antihypertensive medications on measured blood pressure. *Am J Hypertens* 2005; 18(7):935-942.

(46) Fang J, Madhaven S, Alderman MH. Pulse pressure: a predictor of cardiovascular mortality among young normotensive subjects. *Blood Pressure* 2000; 9:260-266.

(47) Zimmermann E, Holst C, Sorensen TI. Lifelong doubling of mortality in men entering adult life as obese. *Int J Obes (Lond)* 2011; 35(9):1193-1199.

(48) Zimmermann E, Holst C, Sorensen TI. Morbidity, including fatal morbidity, throughout life in men entering adult life as obese. *PLoS One* 2011; 6(4):e18546.

(49) Black E, Holst C, Astrup A, Toubro S, Echwald S, Pedersen O et al. Long-term influences of body-weight changes, independent of the attained weight, on risk of impaired glucose tolerance and Type 2 diabetes. *Diabet Med* 2005; 22(9):1199-1205.

(50) Wildman R, Muntner P, Reynolds K, McGinn AP, Rajpathak K, Wyile-Rosett J et al. The obese without cardiometabolic risk factor clustering and the normal weight with cardiometabolic risk factor clustering: prevalence and correlates of 2 phenotypes among the US population (NHANES 1999-2004) . *Arch Intern Med* 2008; 168:1617-1624.

(51) Gordon-Larsen P, Adair LS, Meigs JB, Mayer-Davis E, Herring A, Yan SK et al. Discordant risk: Overweight and cardiometabolic risk in chinese adults. *Obesity (Silver Spring)* 2013; 21(1):E166-E174.

(52) Lawlor DA, Benfield L, Logue J, Tilling K, Howe LD, Fraser A et al. Association between general and central adiposity in childhood, and change in these, with cardiovascular risk factors in adolescence: prospective cohort study. *BMJ* 2010; 341:c6224.

(53) Statland B, Winkel P, Bokelund H. Factors contributing to intra-individual variation of serum constituents: 2. Effects of exercise and diet on variation of serum constituents in healthy subjects. *Clinical Chemistry* 1973; 19:1380-1383.

For peer review only

For peer review only - http://bmjopen.bmj.com/site/about/guidelines.xhtml

STROBE 2007 (v4) Statement—Checklist of items that should be included in reports of *cohort studies*

Section/Topic	Item #	Recommendation	Reported on page #
**Title and abstract**	1	(*a*) Indicate the study's design with a commonly used term in the title or the abstract	1, 2
		(*b*) Provide in the abstract an informative and balanced summary of what was done and what was found	2
**Introduction**			
Background/rationale	2	Explain the scientific background and rationale for the investigation being reported	4
Objectives	3	State specific objectives, including any prespecified hypotheses	5
**Methods**			
Study design	4	Present key elements of study design early in the paper	5
Setting	5	Describe the setting, locations, and relevant dates, including periods of recruitment, exposure, follow-up, and data collection	5
Participants	6	(*a*) Give the eligibility criteria, and the sources and methods of selection of participants. Describe methods of follow-up	5, 6, 7, 8
		(*b*) For matched studies, give matching criteria and number of exposed and unexposed	n/a
Variables	7	Clearly define all outcomes, exposures, predictors, potential confounders, and effect modifiers. Give diagnostic criteria, if applicable	5, 6, 7
Data sources/ measurement	8*	For each variable of interest, give sources of data and details of methods of assessment (measurement). Describe comparability of assessment methods if there is more than one group	5, 6
Bias	9	Describe any efforts to address potential sources of bias	8
Study size	10	Explain how the study size was arrived at	5, 6
Quantitative variables	11	Explain how quantitative variables were handled in the analyses. If applicable, describe which groupings were chosen and why	5, 6, 7, 8
Statistical methods	12	(*a*) Describe all statistical methods, including those used to control for confounding	7, 8
		(*b*) Describe any methods used to examine subgroups and interactions	7, 8
		(*c*) Explain how missing data were addressed	5, 7
		(*d*) If applicable, explain how loss to follow-up was addressed	5, 7
		(*e*) Describe any sensitivity analyses	7, 8
**Results**			

For peer review only

Participants	13*	(a) Report numbers of individuals at each stage of study—eg numbers potentially eligible, examined for eligibility, confirmed eligible, included in the study, completing follow-up, and analysed	5, 8
		(b) Give reasons for non-participation at each stage	5, 8
		(c) Consider use of a flow diagram	n/a
Descriptive data	14*	(a) Give characteristics of study participants (eg demographic, clinical, social) and information on exposures and potential confounders	8
		(b) Indicate number of participants with missing data for each variable of interest	5, 8
		(c) Summarise follow-up time (eg, average and total amount)	5
Outcome data	15*	Report numbers of outcome events or summary measures over time	8
Main results	16	(*a*) Give unadjusted estimates and, if applicable, confounder-adjusted estimates and their precision (eg, 95% confidence interval). Make clear which confounders were adjusted for and why they were included	8, 9, 10
		(*b*) Report category boundaries when continuous variables were categorized	5, 6, 7
		(*c*) If relevant, consider translating estimates of relative risk into absolute risk for a meaningful time period	n/a
Other analyses	17	Report other analyses done—eg analyses of subgroups and interactions, and sensitivity analyses	9, 10
**Discussion**			
Key results	18	Summarise key results with reference to study objectives	9, 10
**Limitations**			
Interpretation	20	Give a cautious overall interpretation of results considering objectives, limitations, multiplicity of analyses, results from similar studies, and other relevant evidence	9, 10, 11, 12
Generalisability	21	Discuss the generalisability (external validity) of the study results	10, 11, 12
**Other information**			
Funding	22	Give the source of funding and the role of the funders for the present study and, if applicable, for the original study on which the present article is based	13

*Give information separately for cases and controls in case-control studies and, if applicable, for exposed and unexposed groups in cohort and cross-sectional studies.

Note: An Explanation and Elaboration article discusses each checklist item and gives methodological background and published examples of transparent reporting. The STROBE checklist is best used in conjunction with this article (freely available on the Web sites of PLoS Medicine at http://www.plosmedicine.org/, Annals of Internal Medicine at http://www.annals.org/, and Epidemiology at http://www.epidem.com/). Information on the STROBE Initiative is available at www.strobe-statement.org.

For peer review only

Online-only supplementary material

Do rapid BMI growth in childhood and early-onset obesity offer cardiometabolic protection to obese adults in mid-life? Analysis of a longitudinal cohort study of Danish men

Laura D Howe*[1], Esther Zimmermann[2], Ram Weiss[3], Thorkild IA Sørensen[2,4]

1 – MRC Integrative Epidemiology Unit at the University of Bristol, School of Social and Community Medicine, University of Bristol, Bristol, UK

2 – Institute of Preventive Medicine, Frederiksberg and Bispebjerg Hospitals, The Capital Region, Copenhagen, Denmark

3 – Braun School of Public Health, The Hebrew University of Jerusalem, Israel

4 - Novo Nordisk Center for Basic Metabolic Research, Faculty of Health and Medical Sciences, University of Copenhagen, Denmark

For peer review only

Details of multilevel model used to estimate childhood BMI trajectories

Differences in size between the participants identified as obese at the draft board examination and the random 1% sample of all individuals were already evident at age seven. A separate intercept and slope for participants identified as obese at the draft board examination was included in the multilevel models in order to model these differences. A linear slope was found to adequately describe BMI changes between seven and 13 years. Therefore two variables summarise each individual's BMI trajectory: an intercept describing their BMI at age seven, and a slope describing their linear change in BMI between seven and 13 years, as defined by the following equation:

$$BMI_{ij} = (\beta0+u_{0j}+e_{0ij}) + (\beta1+u_{1j})(Age_{ij}) + \beta2(Case_{j}) + \beta3(Case*Age_{j}) + e_{ij}(Age_{ij})$$

Where, for individual j at measurement occasion i:

β0=mean estimate of BMI (kg/m^2) at age 7, β1=mean estimates of rate of BMI change between 7-13 years in kg/m^2 per year, β2=mean difference in BMI at age 7 comparing cases (those who were obese in early adulthood, at draft board examination) with non-cases (those not obese at draft board examination), β3=mean difference in rate of BMI change between 7-13 years comparing cases with non-cases, u=individual level random effects, e=error (occasion level random effects)

From the multilevel model, we derived predicted BMI at age 7 and predicted linear change in BMI between 7-13 years (population average intercept and slope, plus the coefficients for mean difference in intercept or slope for those obese at draft board examination if applicable, plus the individual-level residuals for intercept or slope).

For peer review only

Web table 1: Mean (SD) rate of change in BMI across childhood

	Overall	Obese in early adulthood*	Non-obese in early adulthood	P value for difference between obese and non-obese in early adulthood
	N=613	N=329	N=284	
BMI at age 7 (kg/m^2)	17.00 (0.44)	18.50 (2.12)	15.70 (1.18)	<0.001
Rate of BMI growth 7-13yrs (kg/m^2/year)	0.73 (0.44)	1.12 (0.31)	0.40 (0.19)	<0.001

*Defined as 35% overweight relative to a Scandinavian standard in use at the time, equivalent to a body mass index (BMI) ≥31kg/m^2 in early adulthood, mean age 20 years

For peer review only

Web table 2: Model fit for childhood BMI trajectories.

Age	Total number of individuals measured (number who were obese in early adulthood)	Men who were not obese in early adulthood*				Men who were obese in early adulthood			
		Mean (SD) observed BMI	Mean (SD) predicted BMI	Mean difference between observed and predicted measures	95% reference range for the differences between observed and predicted measurements	Mean (SD) observed BMI	Mean (SD) predicted BMI	Mean difference between observed and predicted measures	95% reference range for the differences between observed and predicted measurements
**7 years**	557 (265)	15.71 (1.25)	15.67 (1.21)	+0.04	-0.61 to 0.69	18.50 (2.26)	18.50 (2.11)	+0.004	-1.37 to 1.38
**8 years**	540 (259)	16.18 (1.38)	16.13 (1.26)	+0.04	-0.72 to 0.80	19.71 (2.42)	19.61 (2.15)	+0.10	-1.28 to 1.48
**9 years**	526 (249)	16.54 (1.53)	16.59 (1.38)	-0.04	-0.85 to 0.76	20.65 (2.61)	20.77 (2.31)	-0.12	-1.90 to 1.67
**10 years**	543 (254)	16.96 (1.68)	17.05 (1.55)	-0.09	-1.07 to 0.90	21.80 (2.88)	21.83 (2.43)	-0.03	-2.10 to 2.04
**11 years**	543 (255)	17.40 (1.86)	17.49 (1.69)	-0.09	-1.08 to 0.90	22.93 (2.94)	22.99 (2.58)	-0.07	-2.04 to 1.90
**12 years**	545 (261)	17.93 (1.97)	17.95 (1.83)	-0.02	-0.96 to 0.93	24.20 (3.09)	24.18 (2.80)	+0.02	-2.07 to 2.12
**13 years**	546 (259)	18.61 (2.19)	18.40 (2.01)	+0.21	-0.97 to 1.40	25.31 (3.13)	25.20 (2.97)	+0.11	-2.54 to 2.77

613 participants had at least one or more measure of BMI between 7 and 13 years.

*Defined as 35% overweight relative to a Scandinavian standard in use at the time, equivalent to a body mass index (BMI) ≥31kg/m^2 in early adulthood, mean age 20 years

For peer review only

Web Table 3: Association of obese status in early adulthood and growth in early childhood with metabolic status in mid-life; stratified by mid-life obesity status with adjustment for confounders
Coefficients are odds ratios (95% confidence intervals) comparing obese in early adulthood with non-obese in early adulthood, or a one unit increase in BMI at age 7 or the linear rate of change in BMI between 7 and 13 years.

Model*	Metabolically healthy, normal weight**	Metabolically at risk, normal weight**	Metabolically healthy, obese**	Metabolically at risk, obese**
**Early onset obesity*** **				
3	N=115<br>0.95 (0.47 to 1.94)<br>p=0.90	N=190<br>1 (ref)	N=64<br>0.92 (0.36 to 2.39)<br>p=0.87	N=187<br>1 (ref)
**BMI at 7 (kg/m^2)**				
3	N=116<br>0.95 (0.83 to 1.10)<br>p=0.51	N=188<br>1 (ref)	N=63<br>1.02 (0.89 to 1.17)<br>p=0.81	N=181<br>1 (ref)
**BMI change 7-13 (kg/m^2 per year)**				
3	N=116<br>0.66 (0.23 to 1.91)<br>p=0.44	N=188<br>1 (ref)	N=63<br>1.46 (0.61 to 3.48)<br>p=0.39	N=181<br>1 (ref)

*Model details: Model 3 is adjusted for age at outcome assessment, year of draft board examination, father's social class and current smoking. The coefficient for change in BMI between 7-13 years is additionally adjusted for BMI at 7 years as predicted by the multilevel model
** Metabolically healthy is defined by having pulse pressure (systolic – diastolic blood pressure) <48mmHg, glucose <8.39mmol/l, and cholesterol <6.6mmol/l; i.e. any one or more metabolic abnormality confers 'metabolically at-risk' status. Obesity in mid-life is defined by BMI≥30kg/m^2
***Defined as 35% overweight relative to a Scandinavian standard in use at the time, equivalent to a body mass index (BMI) ≥31kg/m^2 in early adulthood, mean age 20 years

For peer review only

Web Figure 1: Normal plot of individual level residuals for BMI at age 7 from the multilevel model of childhood BMI trajectories

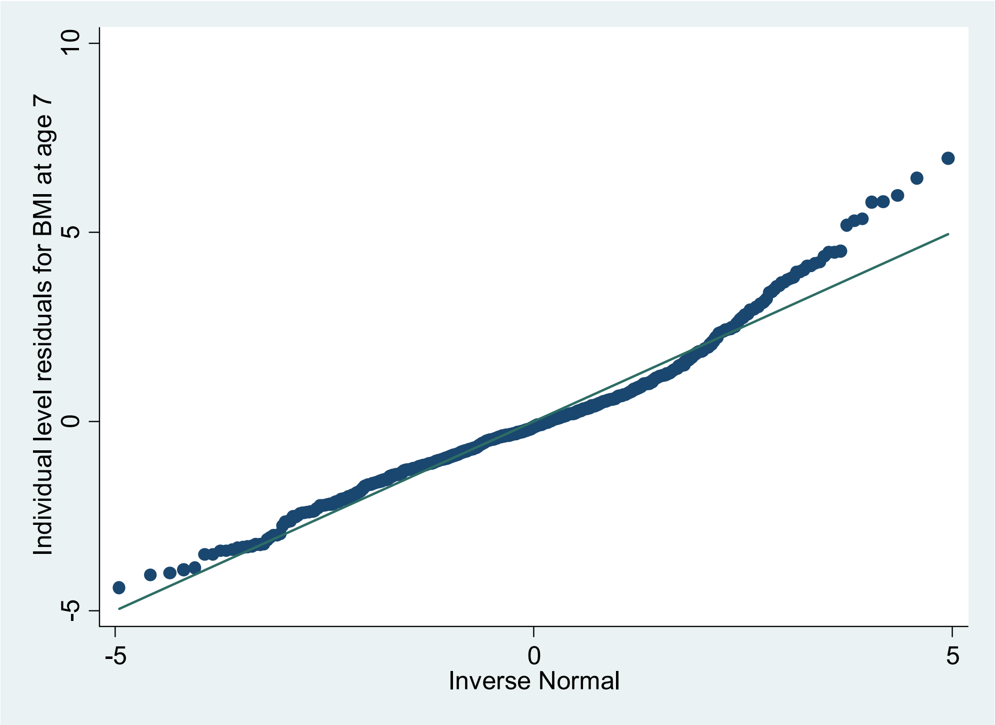

Web Figure 2: Normal plot of individual level residuals for BMI change between 7-13 years from the multilevel model of childhood BMI trajectories

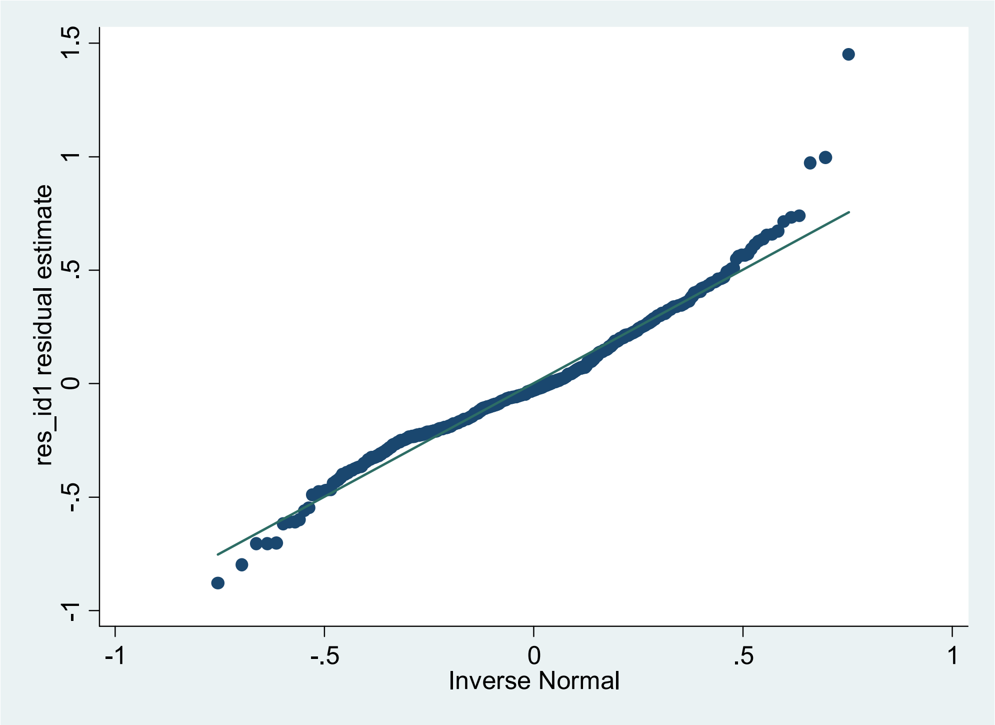

For peer review only

Web Figure 3: Normal plot of occasion level residuals from the multilevel model of childhood BMI trajectories

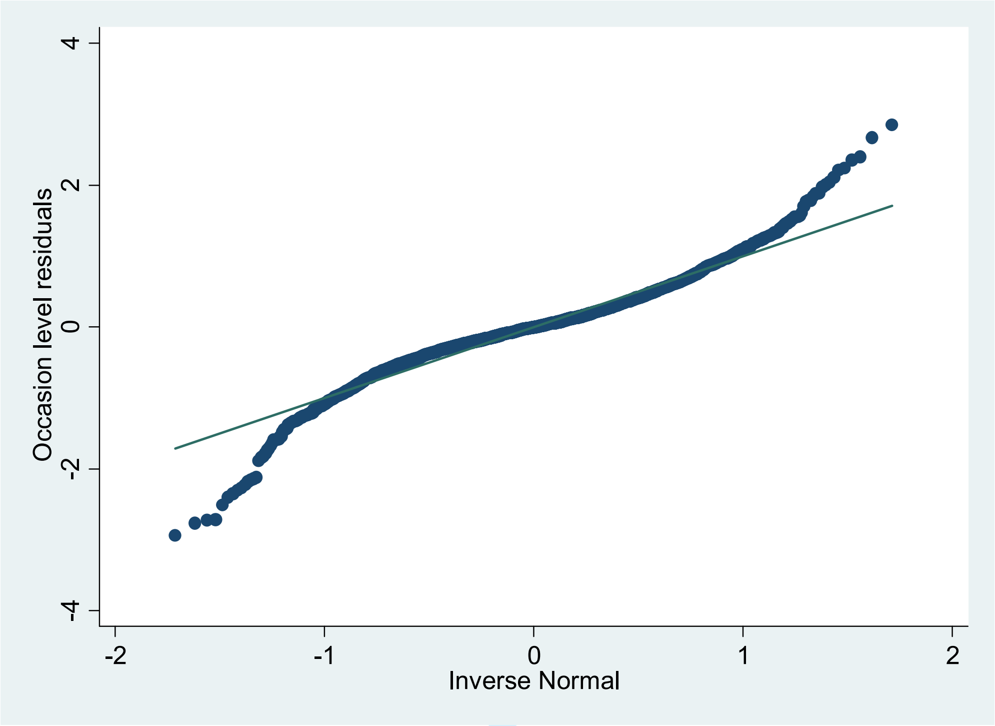

review only

For peer review only
